# A scoping review of multiple deprivation indices in Europe

**DOI:** 10.1093/eurpub/ckaf190

**Published:** 2025-10-30

**Authors:** Gaëlle Mogin, Vanessa Gorasso, Jane Idavain, Maria Lepnurm, Sabrina Delaunay-Havard, Anette Kocbach Bølling, Jurgen Buekers, Axel Luyten, Brecht Devleesschauwer, Carl Michael Baravelli

**Affiliations:** Department of Epidemiology and Public Health, Sciensano, Brussels, Belgium; Department of Epidemiology and Public Health, Sciensano, Brussels, Belgium; Department of Health Statistics, National Institute for Health Development, Tallinn, Estonia; Department of Health Statistics, National Institute for Health Development, Tallinn, Estonia; Environmental and Occupational Health Division, Santé Publique France, Saint-Maurice, France; Department of Air Quality and Noise, Norwegian Institute of Public Health, Oslo, Norway; Centre for Disease Burden, Norwegian Institute of Public Health, Bergen, Norway; Health Unit, Flemish Institute for Technological Research, Mol, Belgium; Department of Epidemiology and Public Health, Swiss Tropical and Public Health Institute, Basel, Switzerland; University of Basel, Basel, Switzerland; Department of Epidemiology and Public Health, Sciensano, Brussels, Belgium; Department of Translational Physiology, Infectiology and Public Health, Faculty of Veterinary Medicine, Ghent University, Merelbeke, Belgium; Department of Disease Burden, Norwegian Institute of Public Health, Bergen, Norway

## Abstract

Multiple deprivation indices (MDIs) measure community-level deprivation using various socio-economic indicators such as education level, unemployment rate, or family structure. With their growing use across Europe and the need to evaluate health impacts on vulnerable populations, this scoping review provides an overview of MDIs in the region. Insights into their construction methods will help provide guidance to researchers in developing future indices. This scoping review was conducted as part of the four-year research project funded through EU Horizon Europe—Burden of disease-based methods for estimating the socio-economic cost of environmental stressors (BEST-COST). We searched Medline, Embase, and Web of Science using terms covering deprivation in Europe. Articles meeting the inclusion criteria were reviewed to identify MDIs and their methodologies. Those including a health indicator were excluded from the study. From 163 articles meeting our inclusion criteria, 18 MDIs were identified. The number of underlying indicators ranged from 4 to 22 across MDIs. Most indices were built for small geographical areas, such as municipalities, districts, or census tracts. Ten indices applied weights derived from statistical methods such as principal components analysis, while the other eight applied equal weights and calculated the index as a simple arithmetic sum or mean composite score. The review highlights high variability in MDI methodologies and emphasizes that aligning MDI selection with the context and objectives of a study. Furthermore, due to the vast cultural and geographical diversity across European countries, developing a Europe-wide index requires careful consideration of the methodologies to be employed.

## Introduction

The concept of an area-based multiple deprivation index (MDI) was first introduced in 1987 by Townsend [1] in the UK to support the targeted allocation of limited resources to areas in greater need. He defined deprivation as “a state of observable and demonstrable disadvantage relative to the local community or the wider society or nation to which an individual, family or group belongs” [1], denoting a phenomenon associated with an accumulation of disadvantages and more complex than poverty. Based on 1981 census data, Townsend combined four indicators (unemployment, household overcrowding, non-home ownership, and non-car ownership) to create a composite score, or index, at the area level. Originally, the Townsend score was used at the level of enumeration districts (EDs) [2], which are geographic areas with an average population of 450, but it can be calculated for any area for which census data are available.

MDIs offer a more complex and multidimensional perspective than that gained by a single measure, such as income or education level and highlight the social aspects of deprivation that are instrumental for health care planning and resources allocation. MDIs are typically measured at the area level to compensate for the lack of individual-level socio-economic data [3]. Area level deprivation, therefore, reflects the socio-economic disadvantage experienced by residents within a specific geographic area, such as a neighbourhood or district.

Since the development of the first MDI by Townsend, several other MDIs have been developed by various other countries, each tailored to a specific geographical resolution. A commonly used MDI in the European context is the European Deprivation Index (EDI) [3–8]. It was initially developed for application in France, but can be applied in 25 additional European countries [9]. Creating an MDI involves multiple stages, requiring researchers to compare various methods and justify their choices at each step. Currently, no comprehensive review offers a methodological framework for selecting appropriate methods based on the study’s context. However, Allik *et al.* [10] identified key factors to consider when developing an MDI such as selecting appropriate data and geographic area, selecting individual deprivation indicators, combining and weighting indicators to construct the index, conducting validation and sensitivity analysis, and dealing with uncertainty.

Based on the growing use of MDIs across Europe and the increasing need to assess health impacts on vulnerable populations, this scoping review aimed to provide a comprehensive overview of MDIs currently applied within the European region, in line with the BEST-COST [11] project’s goal of developing a new framework to assess social inequalities in the health impacts of environmental stressors. Specifically, this scoping review will summarize the MDIs in use, the methodologies guiding their development, and the applied criteria for indicator selection and weighting. The insights gained from examining the variety of methods and approaches to construct these indices will help provide guidance to researchers for developing future MDIs.

## Methods

This scoping literature review was conducted as part of the four-year research project funded through EU Horizon Europe—Burden of disease-based methods for estimating the socio-economic cost of environmental stressors (BEST-COST) [11]. This project aims to improve the methodology, develop and implement an innovative framework for assessing social inequalities in the health impact of environmental stressors using a novel MDI. This literature review was conducted following the guidelines produced by the Centre for Reviews and Dissemination (CRD) [12].

### Data sources and search strategy

We systematically searched Medline, Embase, and Web of Science databases using search terms covering deprivation. The search strategy was developed after consultation with an experienced librarian from the Erasmus MC, The Netherlands in July 2023 and is detailed in Supplementary Additional File 1.

### Inclusion and exclusion criteria

Inclusion and exclusion criteria are summarized in Table 1. We included peer-reviewed studies that used or had developed an MDI, and included a socio-economic dimension such as education, income, or unemployment. In addition, studies were included if they measured an association between deprivation and health outcomes. Only studies conducted in Europe and articles written in one of the national languages of the European Union were considered. Only studies published over the last 10 years have been included (2013–2023) in order to capture MDIs that have been used in a recent context. We excluded studies that did not mention an MDI, those that included an MDI but did not contain a socio-economic dimension, and those that used only one indicator to define deprivation. Studies whose MDI included a health indicator (e.g. health insurance status) were also excluded, as this dimension is part of the outcomes assessed under the BEST-COST project, i.e. burden of disease measures. Incorporating a health indicator into a deprivation index when analyzing health outcomes raises methodological concerns, as it could introduce bias by partially reflecting the very outcome it aims to predict.

**Table 1.** Table summarizing inclusion and exclusion criteria

	Inclusion	Exclusion
Population	European studies (conducted in Europe and written in a national EU country language)	Non-European studies
Outcomes	MDI is used in conjunction with a health outcome in the study	No health outcome included in the analysis
Study design	Include an MDI that integrates a social dimension	MDI does not include a social dimension, or single indicator is used to define deprivation. MDI includes a health indicator
Time frame	2013–2023	Before 2013

### Screening and data extraction

The publications were screened using the Rayyan [13] web tool for systematic reviews. Three researchers (G.M., C.B., and V.G.) independently examined the results of the search. A first screening was carried out on the basis of title and abstract, followed by a second screening examining full texts. Potential disagreements were resolved by a meeting between the three researchers. The extraction items for data extraction were previously discussed with input from the broader BEST-COST project team and focused on MDI-related methodologies. Definitions for these items are provided in Supplementary Additional file 2. Data extraction was then performed independently by five researchers (G.M., C.B., V.G., J.I., and M.L.) using two spreadsheets. The first one contains details on each selected study, including PMID/DOI, first author name, year, journal name, title of paper, study region, target population, index used, environmental exposure, health outcome, use of the index (development, validation) and additional notes (see Supplementary Additional file 2). The second spreadsheet provides information on each reported MDI, such as the indicators included in the index, the data source for each indicator, the reference year of the data source, the geographic scale applied, the average population size of the geographical scale, the method used for selecting the indicators and whether weights were used, the method for weight computation, uncertainty assessment, and additional notes.

Geographical scale refers to the level of spatial detail or extent at which geographic data are analysed or represented. It ranges from broad, large-scale perspectives that cover extensive areas (e.g. regions, provinces, countries) to more localized, small-scale views that provide greater detail (e.g. municipalities, districts).

Weights in the context of MDIs refer to the assigned values that reflect the relative importance of different underlying indicators used to measure deprivation. This process prioritizes certain indicators over others and can be guided by expert opinion or derived through statistical methods such as principal components analysis (PCA) or factor analysis (FA). A common approach is to simply apply equal weights to each indicator. In MDI weighting, PCA is a statistical method that identifies underlying principal components from a list of indicators based on the coefficient matrix, which indicates the contribution of each original indicator to the principal components.

## Results

### Study selection

**Figure 1.**
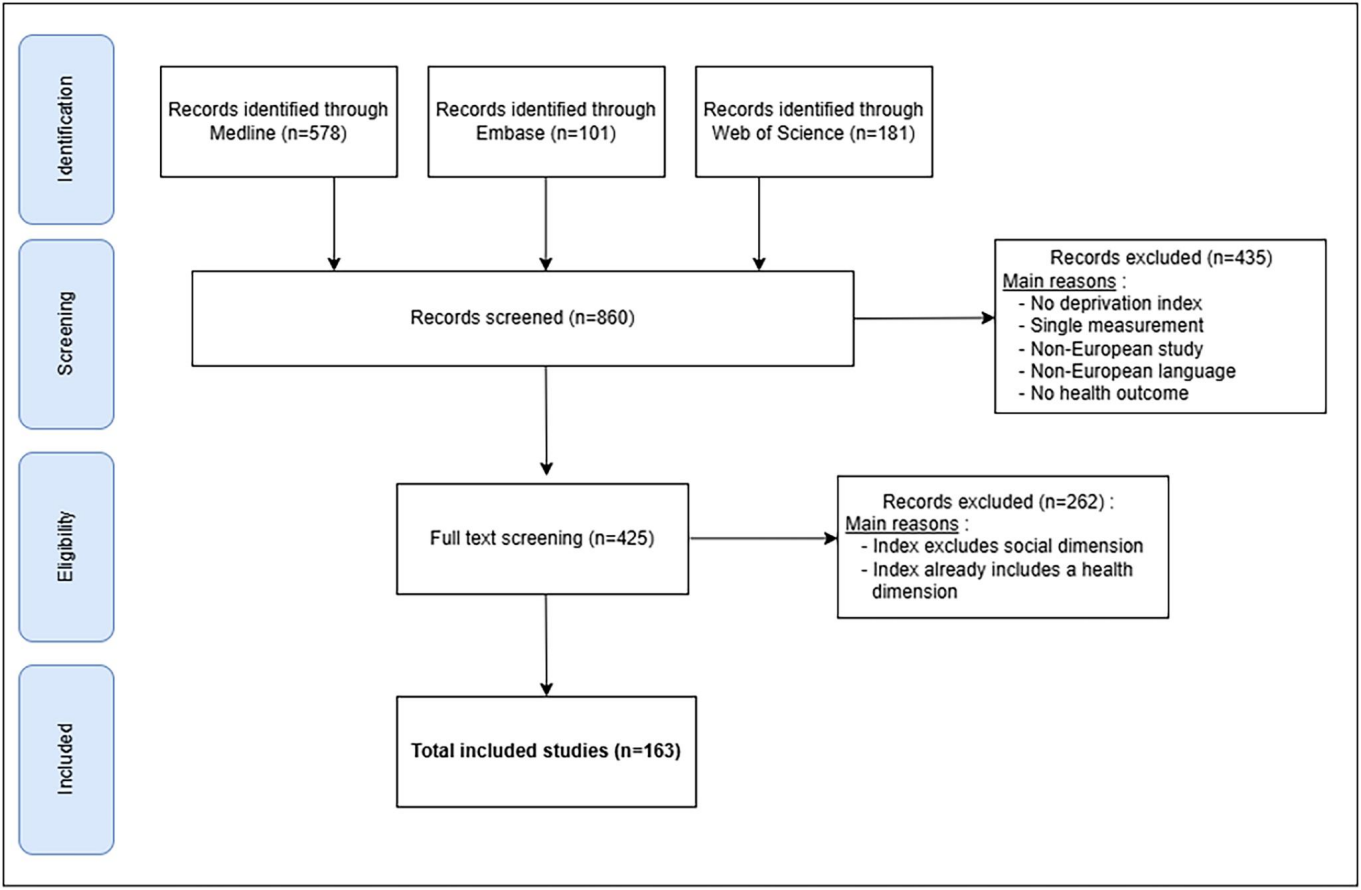
PRISMA flowchart of MDI scoping review.

The database searches resulted in 860 records after elimination of duplicates. The records were then imported into the Rayyan [13] platform for selection based on title and abstract, using predefined inclusion and exclusion criteria. Following this screening, 425 studies were chosen for full-text review and assessed for eligibility. In total, 163 articles met the inclusion criteria (Fig. 1—PRISMA flowchart).

### Indices diversity and methodologies

Following data extraction, we identified 18 indices of multiple deprivation. The number of articles per MDI and their details can be found in Supplementary Additional File 3. Over the past decades, the European Deprivation Index emerged as the most cited index, with 33 records (see Supplementary Additional File 3), followed by the EPICES score (France; 20 records) and the Townsend Deprivation Index (UK; 17 records). The remaining 15 indices were used in 13 or fewer studies.

General information such as data source, geographical level, and weighting methods of these indices is summarized in Table 2. The extracted MDIs were primarily developed for or used in a variety of European regions, including numerous instances in France, Italy, the UK, and Ireland, with additional applications in Portugal, Spain, Slovenia, Russia, and Germany.

**Table 2.** General information on the 18 extracted indices, including the index name and acronym, the country of origin, the type of data source, the geographical level of application, whether differential weighting was used (and the method employed, if applicable), and the number of indicators in the original index.

Name of the index (acronym)	Original country	Data source of the specific indicator	Geographical scale	Use of weights in the index formula	Method for computation of weights	Number of indicators in the index
Area Based Deprivation Index (ABDI)	Spain	Census	Census section, neighbourhoods, districts	No	NA^a^	7
Bavarian Index of Multiple Deprivation (BIMD)	Germany	Administrative data	District	Yes	PCA	7
Carstairs score	UK	Census	Postcode sector	No	NA	4
Child Material and Social deprivation	EU	Survey data	Country (EU-28)	No	NA	17
Danish Deprivation Index (DANDEX)	Denmark	Administrative data	Parish level	Yes	PCA	9
EPICES score	France	Survey	District	Yes	PCA	11
European Deprivation Index (EDI)	France	Survey data, census data	Census tract and lower super output areas (Portugal and England); municipal level for other countries	Yes	Multivariate logistic regression; weights are constructed individually for each country	10^b^
French Deprivation Index (FDep)	France	Census	IRIS level^c^	Yes	PCA	4
German Index of Multiple Deprivation (GIMD)	Germany	Census, administrative data	Municipality	Yes	PCA	9
German Index of Socio-economic Deprivation (GISD)	Germany	Administrative data	Municipality	Yes	PCA	9
Irish National Deprivation Index	Ireland	Census	Electoral Divisions	Yes	PCA	4
Italian Deprivation Index (DI)	Italy	Census	Municipality	No	NA	5
Neighbourhood Deprivation Index	France	Census	Metropolitan areas (Lille, Lyon, Paris)	No	NA	22
Pobal HP Deprivation Index	Ireland	Registry	District electoral division (DED)	Yes	Confirmatory factor analysis	10
Russian deprivation index	Russia	Census	Region	Yes	PCA	17
Socio-Economic and Health-related Deprivation Index (SEHDI)	Italy	Census	Municipality, region	No	NA	14
SoDep Index	EU	Survey	Country	Yes	Standardized structural equation modeling (SEM)	6
Townsend Deprivation index (TDI)	UK	Census	Enumeration district	No	NA	4

a NA = not applicable.

b The original EDI constructed in France includes 10 indicators but this number varies in other countries that used the EDI.

c IRIS : “Ilots regroupés pour l'information statistique” = grouped blocks for statistical information.

The number of underlying indicators in each index varied significantly, ranging from 4 (several indices) to 22 (neighbourhood deprivation index; France). The most applied data source was census data (*n* = 11 indices, 61%), while administrative data (*n* = 4, 22%) or survey data (*n* = 4, 22%) were also used.

The most often used geographical scales in the indices recorded were based on municipalities, census tracts, and districts or equivalent geographic levels such as parishes or electoral divisions. Some indices were designed for use at even finer geographical scales. For instance, the child material and social deprivation index was applied at the statistical sector level (small census tract), while the FDep index (French Deprivation index) was used at the IRIS level (small divisions that partition France into approximately 15 500 sectors) [14].

With regard to the application of weights in the development of the MDI, slightly more than half of the included indices (*n* = 10, 55%) used differential weights when combining the indicators into a single composite score while the other eight opted for equally weighted indicators. The most common method used to estimate weights was PCA (*n* = 7/10 indices that used differential weights, 70%). Other methods that were reported for developing weights included the use of the beta coefficients derived from regression models, factor scores derived from FA and standardized structural modeling (SEM).

### Diversity of underlying indicators

**Figure 2.**
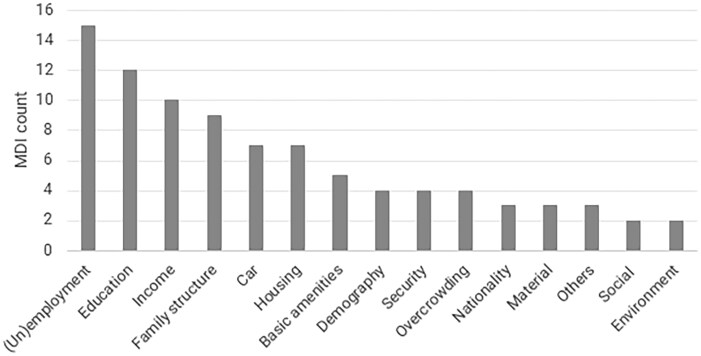
Number of indices applying underlying indicators from each domain (the total number of indices is 18). Note that some indices include more than one underlying indicator from one domain.

The number and type of indicators used differ significantly across indices. The indicators were categorized by domain (or dimension), as shown in Fig. 2. Certain domains were more frequently utilized than others, such as (un)employment/occupation (15 indices out of 18, 83%), education (*n* = 12, 67%), income (*n* = 10, 56%), and family structure (*n* = 9, 50%). A comprehensive list of the extracted underlying indicators is available in Supplementary Additional File 4. The indicator used to define a domain (e.g. education) also varied widely between indices. For instance, while DANDEX (Denmark) defined education level as “the proportion of inhabitants with a basic education”, FDep (France) considers “the percentage of high school graduates,” and Pobal HP (Ireland) focuses on “the percentage of inhabitants with only a primary school education”.

It is important to note that in several cases, multiple indicators within the same domain are included in one index. For example, the BIMD (Germany) considers both “the percentage of residents in financial poverty” and “the financial situation of districts” in the income domain.

## Discussion

This scoping literature review aimed to compile European studies that developed or used an MDI between 2013 and 2023 in order to guide the development of a European index for the BEST-COST project. A key objective of this project is to evaluate the impact of air and noise pollution on non-communicable diseases while accounting for deprivation across diverse case-study countries, specifically Belgium, Estonia, France, Norway, and Portugal. The development of an MDI for this project requires compliance with specific criteria, such as the availability of data—preferably on a small area level—relevant indicators and the achievement of a level of reproducibility that applies both to the countries studied and can be extended to other countries.

Information on application of MDIs, as well as data sources and methodological choices required to develop the indices was extracted from a total of 163 articles. Eighteen MDIs meeting our criteria were identified and applied across Europe. They varied notably in frequency of use and their development methodologies. In particular, the definition and number of underlying indicators included in the indices ranged widely (from 4 to 22). Despite this diversity, several common methodological approaches emerged, such as the frequent use of census data as a primary source, the application of MDIs at a small geographical level, and the use of either simple equal weighting or PCA for weight assignment.

The geographical scale used in constructing an MDI is crucial due to the influence of environmental, cultural, and data availability factors. MDIs, which aggregate socio-economic characteristics at the area level, aim to capture the multidimensional nature of deprivation experienced by its residents. In our study, we observed that nearly all indices were applied at a small geographical level, although the spatial resolution differed based on the study and country. The most commonly recorded scales included municipalities, census sections, districts, or comparable geographic units such as parishes (Denmark, Portugal) or electoral divisions (Ireland). Additionally, some indices were tailored for application at even smaller geographical scales.

On a national level, analysing deprivation over smaller geographical areas helps to identify localized deprivation and potentially develop targeted policies for the most disadvantaged regions. Although national-level indices provide a standardized measure across a country, they present certain challenges such as assuming uniform deprivation determinants, which may not reflect the unique socio-economic and cultural characteristics of diverse regions [3]. Some studies also suggest that national indices are more suited for urban areas, as their component indicators often align with urban settings, whereas rural areas may require specialized indices to capture their specific conditions [3, 15–17]. For example, the French Ecological Deprivation Index (F-EDI) [3] demonstrated that, despite national-level definitions, it effectively identified deprivation across various areas of Metropolitan France. However, a more localized approach may be necessary when investigating specific health inequalities in particular regions. The geographical scale also influences how deprivation affects outcomes. An English study on breast cancer survival showed that larger geographic areas diluted deprivation differentials, as the larger populations led to greater social heterogeneity [18]. Similarly, in Quebec, regional and local versions of the MDI were developed to address the needs of various users and improve the analysis of health inequalities across 15 metropolitan areas [19]. Thus, it is essential to consider the unique characteristics of each country and the context of the study before selecting the geographical scale for developing or applying the MDI.

The variation in the definition and number of indicators identified in each index we found can partly be explained by the fact that the MDIs were developed to meet different needs and, in some cases, were tailored to specific target populations. For instance, the “Child Material and Social Deprivation” [20] index (EU index) focuses on children, while the SoDep index (EU index) has been applied to the elderly [21]. While a literature review can guide indicator selection, the inclusion of specific indicators often hinges on data availability within a region or country. Therefore, it is advisable to assess data availability at this stage, and maybe conducting a pilot study for data collection. Statistical techniques such as PCA can be used to reduce the number of indicators.

Cultural and societal differences across European countries are another factor that can contribute to the variability in inclusion of underlying indicators in the indices. The different weighting assigned to the indicators of five different countries (France, Italy, Portugal, Spain, and England) for the implementation of the EDI [4] indicates that the indicators do not have the same importance from one country to another. For example, the weight (regression model coefficients) given to the “low-income occupations” indicator varies significantly: .57 for France, .19 for Italy, .01 for Portugal, .62 for Spain, and .39 for England. However, the EDI study also shows that despite these differences, the similarities observed between countries at different stages of development suggest that cultural differences may have a smaller impact than expected. This is because the basic needs of populations across the five countries are relatively similar. As a result, the EDI demonstrates that it is possible to create an MDI with common indicators across countries, while still accounting for country-specific weighting of those indicators [4]. Nevertheless, the study states that it would be interesting to carry out exploratory studies on the impact of heterogeneity in the size of geographical zones on comparability between countries. Another study on the English Index of Multiple Deprivation (IMD) [22] emphasized that analyses of inequalities, both between and within countries, by socio-economic position should use consistent measures. Failing to do so could lead to misleading results.

We observed that, regarding weighting methods, 10 out of 18 studies applied differential weights, while seven studies assigned equal weights to all indicators. Using equal weights has both advantages and disadvantages. Although it does not account for the relative importance of individual indicators, equal weighting can still be applied even in the absence of sufficient understanding of causal relationships or agreement on an alternative approach [23]. It also simplifies implementation and facilitates comparability of deprivation across countries. Since the BEST-COST project involves five geographically and culturally diverse European countries, the creation of an index with equal weights is worth considering. Commonly used indices, such as the Townsend Index [1] and the Carstairs Score [24], are examples of measures that apply an equal weights approach. In addition, a study conducted on the German Index of Multiple Deprivation (GIMD) [25] with four weighting scenarios demonstrated that the association between area deprivation and mortality proved to be stable and showed rather small differences, regardless of the weighting method. However, despite the strong correlation observed, the study suggests that using equal domain weighting may be outdated, as it effectively results in an implicit weighting driven by the availability of indicators within each domain. Therefore, it is recommended to carefully consider the study’s context and objectives when determining whether to apply weights to the indicators.

A major challenge in constructing an MDI lies in the fact that it inherently involves subjectivity due to choices regarding variables, weights, and geographical scale. Despite their widespread use in public health, MDIs are rarely extensively validated, primarily due to the complexity of the process [3, 19]. Validating an MDI involves determining whether it accurately reflects deprivation, which requires meeting specific criteria and properties relevant to its application [3, 19, 26, 27].

### Strengths and limitations

Our literature review compiles existing studies on the development and application of various multiple deprivation indices across Europe. It provides a detailed examination of the methodological approaches and assumptions employed in research linking deprivation to health outcomes. The review draws on three search engines and benefits from collaboration with public health experts from multiple European countries. However, our study has a number of limitations. In particular, it is important to note that not all MDIs in Europe are included in this review, since we chose to exclude indices that included a health component, as well as indices that did not incorporate a social component. As a result, several important and widely used indices such as the English Index of Multiple Deprivation [28] and its derivates have not been detailed in this study. Furthermore, contrary to what is generally done in systematic literature reviews, we did not perform a quality assessment of the included studies. Since our focus was on extracting methodological information, we did not consider an assessment of bias to be relevant to the aims of this literature review.

### Research implications

This study offers guidance for creating a new multiple deprivation index, based on a comprehensive review of existing European MDIs. It also provides input to the BEST-COST project that will quantify material and social deprivation associated with exposure to air pollution and noise across the five case countries studied: Belgium, Estonia, France, Norway, and Portugal.

## Conclusions

In this literature review, we examined independent studies that have used or developed a multiple deprivation index in European countries. We observed considerable variation in the methodologies employed across these indices. While most studies use a small geographic scale, there is significant variability in the definition and number of selected indicators, as well as in the data sources. The decision whether or not to apply weights is also crucial and should be guided by the context and objectives of the study. It is important to note that few indices have been validated, meaning they should be used with caution. Analysing these methodologies helps inform the development of a multiple deprivation index for the BEST-COST project, as well as other future MDI.

## Supplementary Material

ckaf190_Supplementary_Data

## Data Availability

The authors confirm that the data supporting the findings of this study are available within the article and its supplementary materials. Key points Cultural and societal differences across European countries play a key role in driving MDIs methodological diversity. Factors such as data availability, study context, and objectives must be considered, as they shape essential methodological choices. A core challenge in constructing an MDI stems from its inherent subjectivity, arising from choices related to indicators, weighting, and geographical scale. There is a need to conduct more validation studies on MDIs and to investigate issues such as the potential impact of the heterogeneity of the geographical areas explored.
