# Supplementary material for: A scoping review of multiple deprivation indices in Europe
Source: Eur J Public Health. 2025 Oct 30;35(6):1122–8. doi: 10.1093/eurpub/ckaf190 (PMC12707476; doi:10.1093/eurpub/ckaf190)
Supplement: ckaf190_Supplementary_Data [file ckaf190_supplementary_data.zip › ejph-2025-01-om-0031-File004.docx]

**Additionnal file 1 : search strategy**

| **Database searched** | **Platform** | **Years of coverage** | **Records** | **Records after duplicates removed** |
| --- | --- | --- | --- | --- |
| Medline ALL | Ovid | 1946 - Present | 586 | 578 |
| Embase | Embase.com | 1971 - Present | 535 | 101 |
| Web of Science Core Collection* | Web of Knowledge | 1975 - Present | 480 | 181 |
| **Total** | | | **1601** | **860** |

*Science Citation Index Expanded (1975-present) ; Social Sciences Citation Index (1975-present) ; Arts & Humanities Citation Index (1975-present) ; Conference Proceedings Citation Index- Science (1990-present) ; Conference Proceedings Citation Index- Social Science & Humanities (1990-present) ; Emerging Sources Citation Index (2005-present)

No other database limits were used than those specified in the search strategies

**Embase 535**

('deprivation index'/de OR 'social deprivation index'/de OR 'Townsend deprivation index'/mj/de OR 'socioeconomic deprivation'/mj/de OR (('social isolation'/mj/de OR 'social inequality'/mj/de) AND (indicator/de OR 'health status indicator'/de)) OR (((deprivat* OR socioecomic* OR socio-economic* OR soc*-ineq*) NEAR/12 (index* OR indices OR measure* OR indicator*)) OR ((socioecomic* OR socio-economic* OR social* OR econom*) NEAR/3 (deprivat*)) OR socio-index* OR social-index*):ti,kw) **AND** ('Europe'/exp OR 'Yugoslavia'/de OR 'Israel'/de OR 'European Union'/de OR 'European'/de OR 'EU citizen'/de OR Cyprus/de OR 'Turkey (republic)'/de OR (europ* OR austria* OR belgium OR belgian* OR Denmark OR danish OR france OR french* OR german* OR ireland OR irish* OR italy OR italian* OR luxemb* OR netherlands OR dutch OR norway OR sweden OR switzerland OR swiss OR united-kingdom OR albania OR armenia OR bosnia* OR herzegovin* OR bulgar* OR croatia* OR cyprus OR czechoslovakia* OR estonia* OR finland OR georgia OR greece OR hungar* OR iceland* OR israel* OR kosov* OR latvia* OR lithuan* OR macedoni* OR malta OR montenegr* OR poland OR polish OR portug* OR romani* OR rumani* OR serbi* OR slovak* OR sloven* OR spain* OR spanish OR turkey* OR mediterran* OR czech* OR england* OR UK OR scotland OR wales OR britain* OR holland* OR scandinav* OR nordic-countr* OR yugoslov* OR baltic* OR flander* OR wallon* OR benelux* OR greek* OR andorra* OR azerbaijan* OR belarus* OR byelarus* OR byelorus* OR russia* OR monaco* OR moldova* OR moldovia* OR san-marin* OR ukrain*):ab,ti,kw,jt) AND [2013-2030]/py NOT ([Conference Abstract]/lim OR [Conference Review]/lim)

**Medline 586**

(*Social Deprivation/ OR ((*Social Isolation/ OR *Socioeconomic Factors/) AND (Health Status Indicators/)) OR (((deprivat* OR socioecomic* OR socio-economic* OR soc*-ineq*) ADJ12 (index* OR indices OR measure* OR indicator*)) OR ((socioecomic* OR socio-economic* OR social* OR econom*) ADJ3 (deprivat*)) OR socio-index* OR social-index*).ti,kf.) **AND** (exp Europe/ OR exp Yugoslavia/ OR exp Israel/ OR exp European Union/ OR exp European People/ OR Turkey/ OR (europ* OR austria* OR belgium OR belgian* OR Denmark OR danish OR france OR french* OR german* OR ireland OR irish* OR italy OR italian* OR luxemb* OR netherlands OR dutch OR norway OR sweden OR switzerland OR swiss OR united-kingdom OR albania OR armenia OR bosnia* OR herzegovin* OR bulgar* OR croatia* OR cyprus OR czechoslovakia* OR estonia* OR finland OR georgia OR greece OR hungar* OR iceland* OR israel* OR kosov* OR latvia* OR lithuan* OR macedoni* OR malta OR montenegr* OR poland OR polish OR portug* OR romani* OR rumani* OR serbi* OR slovak* OR sloven* OR spain* OR spanish OR turkey* OR mediterran* OR czech* OR england* OR UK OR scotland OR wales OR britain* OR holland* OR scandinav* OR nordic-countr* OR yugoslov* OR baltic* OR flander* OR wallon* OR benelux* OR greek* OR andorra* OR azerbaijan* OR belarus* OR byelarus* OR byelorus* OR russia* OR monaco* OR moldova* OR moldovia* OR san-marin* OR ukrain*).ab,ti,kf,jw) AND 2013:2030.(sa_year). NOT (news OR congres* OR abstract* OR book* OR chapter* OR dissertation abstract*).pt.

**Web of Science 480**

TI=(((deprivat* OR socioecomic* OR socio-economic* OR soc*-ineq*) NEAR/12 (index* OR indices OR measure* OR indicator*)) OR ((socioecomic* OR socio-economic* OR social* OR econom*) NEAR/2 (deprivat*)) OR socio-index* OR social-index*) **AND** TS=(europ* OR austria* OR belgium OR belgian* OR Denmark OR danish OR france OR french* OR german* OR ireland OR irish* OR italy OR italian* OR luxemb* OR netherlands OR dutch OR norway OR sweden OR switzerland OR swiss OR united-kingdom OR albania OR armenia OR bosnia* OR herzegovin* OR bulgar* OR croatia* OR cyprus OR czechoslovakia* OR estonia* OR finland OR georgia OR greece OR hungar* OR iceland* OR israel* OR kosov* OR latvia* OR lithuan* OR macedoni* OR malta OR montenegr* OR poland OR polish OR portug* OR romani* OR rumani* OR serbi* OR slovak* OR sloven* OR spain* OR spanish OR turkey* OR mediterran* OR czech* OR england* OR UK OR scotland OR wales OR britain* OR holland* OR scandinav* OR nordic-countr* OR yugoslov* OR baltic* OR flander* OR wallon* OR benelux* OR greek* OR andorra* OR azerbaijan* OR belarus* OR byelarus* OR byelorus* OR russia* OR monaco* OR moldova* OR moldovia* OR san-marin* OR ukrain*) AND py=(2013-2030) NOT DT=(Meeting Abstract OR Meeting Summary)
