# Supplementary material for: A scoping review of multiple deprivation indices in Europe
Source: Eur J Public Health. 2025 Oct 30;35(6):1122–8. doi: 10.1093/eurpub/ckaf190 (PMC12707476; doi:10.1093/eurpub/ckaf190)
Supplement: ckaf190_Supplementary_Data [file ckaf190_supplementary_data.zip › ejph-2025-01-om-0031-File005.docx]

**Additional file 2 - definitions of the data extraction items**

**Sheet 1 : study information**

| General information | PMID or DOI | A unique identifier number assigned to a specific reference/article |
| --- | --- | --- |
|  | First author | Name of the first author of the article |
|  | Year | The year that the selected study was published |
|  | Journal | The name of the journal that published the selected article |
|  | Title of paper | Title of the selected article |
| Study characteristics | Country/region studied | The country or region of the study. Several countries or regions can be indicated |
|  | Population included (overall population, children,..) | Population targeted in the study. This could be the general population or specific groups such as children, the elderly,.. |
| Index used | Name of the index used | Name of the multiple deprivation index used in the article and its acronym |
| Content | Health outcome | Name of the health outcome targeted in the study |
|  | Environmental exposure included | Indicates whether an environmental exposure is measured in conjunction with deprivation in the article, and if so, which one it is (e.g. chemical exposure). |
|  | Content (index development, revision, validation,..) | Indicates the use of the index in the article, i.e. whether the article discusses its development, validation, use or revision. |
| Additional information | Additional notes | Indicates additional information that may be required (e.g. a link to another study providing more details on MDI). |

**Sheet 2 : indicators and MDI information**

| General information | Name of the index + acronym | Name of the multiple deprivation index used in the article and its acronym |
| --- | --- | --- |
| Indicator | Indicators (education level, income,..) | Name of the indicator used to construct the MDI (e.g. unemployment rate in the population, average income of the population, etc.). One indicator is indicated per line, the aim being to indicate all the indicators present in the same index. |
| Data information | Data source of the specific indicator | Name of the indicator data source (interview, census, questionnaire, examination, etc.) |
|  | Reference year | Indicator data reference year |
| Geographical scale | Geographical scale (municipality, region,..) | Name of geographic scale of indicator used, e.g. municipality, region, statistical area, postal code |
|  | Average population size of the geographical scale | Average population size of the geographic scale. This can be in numbers of people, households or patients, for example. |
| Methods | Method for selecting the indicators | Method of selecting the indicator to compose the MDI, e.g. PCA, FA or expert opinion |
|  | Standardisation method | Name of indicator standardization method (if any) e.g. z-score method, regression coefficients |
|  | Whether weights were used or not | Indicates whether weights have been assigned to each indicator. Weights in the context of MDIs refer to the assigned values that reflect the relative importance of different indicators used to measure deprivation. Weights prioritise certain indicators over others based on their significance in affecting the defining of deprivation. |
|  | Method for weight computation | Name of the method used to assign a weight to each indicator, e.g. PCA, FA, multivariate logistic regression |
| Uncertainty | Uncertainty assessment | Indicates whether uncertainty has been assessed |
| Additional information | Additional notes | Indicates additional information that may be required (e.g. more details on the method used to compute the MDI). |
