# Supplementary material for: A scoping review of multiple deprivation indices in Europe
Source: Eur J Public Health. 2025 Oct 30;35(6):1122–8. doi: 10.1093/eurpub/ckaf190 (PMC12707476; doi:10.1093/eurpub/ckaf190)
Supplement: ckaf190_Supplementary_Data [file ckaf190_supplementary_data.zip › ejph-2025-01-om-0031-File006.docx]

**Additional file 3 : references per MDI**

| **Name of the index (acronym)** | **Reference** |
| --- | --- |
| Area Based Deprivation Index (ABDI) | ^1^ |
| Bavarian Index of Multiple Deprivation (BIMD) | ^2–4^ |
| Carstairs score | ^5–17^ |
| Child Material and Social deprivation | ^18,19^ |
| Danish Deprivation Index (DANDEX) | ^20^ |
| EPICES* score | ^21–40^ |
| European Deprivation Index (EDI) | ^41–73^ |
| French Deprivation Index (FDEP) | ^38,74–86^ |
| German Index of Multiple Deprivation (GIMD) | ^4,87–91^ |
| German Index of Socioeconomic Deprivation (GISD) | ^92–95^ |
| Irish National Deprivation Index | ^96–104^ |
| Italian Deprivation Index (DI) | ^105–114^ |
| Neighbourhood Deprivation Index | ^115–119^ |
| Pobal HP Deprivation Index | ^120–123^ |
| Russian deprivation index (RDI) | ^124^ |
| Socio-Economic and Health-related Deprivation Index (SEHDI) | ^110,125–131^ |
| SoDep Index | ^132^ |
| Townsend Deprivation Index (TDI) | ^133–149^ |
| Other* | ^150–163^ |

* The “other” category includes publications that discuss multiple indices without applying them, or where the details of the index are not clearly specified.

Figure showing the number of articles per index in publications from 2013 to 2023. The eight most commonly applied indices are displayed as individual bars, while the remaining ten are combined under ‘Others’. EDI = European Deprivation Index, EPICES (French index) = « Evaluation de la précarité et des inégalités de santé dans les Centres d'examens de santé », Fdep = French deprivation index, IDI = Italian Deprivation Index, INDI = Irish National Deprivation Index, SEHDI = Socio-Economic and Health-related Deprivation Index. The “Others” category comprises the remaining 10 indices.

**References**

1. Cebrecos A, Domínguez-Berjón MF, Duque I, Franco M, Escobar F. Geographic and statistic stability of deprivation aggregated measures at different spatial units in health research. Appl Geogr. 2018 Jun 1;95:9–18.

2. Beyerlein A, Koller D, Ziegler AG, Lack N, Maier W. Does charge-free screening improve detection of gestational diabetes in women from deprived areas: a cross-sectional study. BMC Pregnancy Childbirth. 2016 Sep 9;16(1):266. doi: 10.1186/s12884-016-1060-3. PMID: 27613387; PMCID: PMC5016952.

3. Manz KM, Schwettmann L, Mansmann U, Maier W. Area Deprivation and COVID-19 Incidence and Mortality in Bavaria, Germany: A Bayesian Geographical Analysis. Front Public Health. 2022 Jul 15;10:927658. doi: 10.3389/fpubh.2022.927658. PMID: 35910894; PMCID: PMC9334899.

4. Maier W. Indizes Multipler Deprivation zur Analyse regionaler Gesundheitsunterschiede in Deutschland : Erfahrungen aus Epidemiologie und Versorgungsforschung [Indices of Multiple Deprivation for the analysis of regional health disparities in Germany : Experiences from epidemiology and healthcare research]. Bundesgesundheitsblatt Gesundheitsforschung Gesundheitsschutz. 2017 Dec;60(12):1403-1412. German. doi: 10.1007/s00103-017-2646-2. Erratum in: Bundesgesundheitsblatt Gesundheitsforschung Gesundheitsschutz. 2017 Dec;60(12):1455-1456. doi: 10.1007/s00103-017-2664-0. PMID: 29119206.

5. Allik M, Brown D, Dundas R, Leyland AH. Small-area deprivation measure datasets for Scotland, 2001 and 2011. Data Brief. 2016 Apr 30;7:1682-1686. doi: 10.1016/j.dib.2016.04.060. PMID: 27761509; PMCID: PMC5063812.

6. Allik, Mirjam & Brown, Denise & Dundas, Ruth & Leyland, Alastair. (2016). Developing a new small-area measure of deprivation using 2001 and 2011 census data from Scotland. Health & Place. 39. 122-130. 10.1016/j.healthplace.2016.03.006.

7. Almendra R, Santana P, Vasconcelos J. Evidence of social deprivation on the spatial patterns of excess winter mortality. Int J Public Health. 2017 Nov;62(8):849-856. doi: 10.1007/s00038-017-0964-7. Epub 2017 Mar 30. PMID: 28361292; PMCID: PMC5641277.

8. Bertin M, Chevrier C, Pelé F, Serrano-Chavez T, Cordier S, Viel JF. Can a deprivation index be used legitimately over both urban and rural areas? Int J Health Geogr. 2014 Jun 14;13:22. doi: 10.1186/1476-072X-13-22. PMID: 24929662; PMCID: PMC4063986.

9. Bryere J, Pornet C, Copin N, Launay L, Gusto G, Grosclaude P, Delpierre C, Lang T, Lantieri O, Dejardin O, Launoy G. Assessment of the ecological bias of seven aggregate social deprivation indices. BMC Public Health. 2017 Jan 17;17(1):86. doi: 10.1186/s12889-016-4007-8. PMID: 28095815; PMCID: PMC5240241.

10. Chen R, McKevitt C, Rudd AG, Wolfe CD. Socioeconomic deprivation and survival after stroke: findings from the prospective South London Stroke Register of 1995 to 2011. Stroke. 2014 Jan;45(1):217-23. doi: 10.1161/STROKEAHA.113.003266. Epub 2013 Nov 21. PMID: 24262326.

11. Clement ND, Duckworth AD, Wickramasinghe NR, Court-Brown CM, McQueen MM. Does socioeconomic status influence the epidemiology and outcome of distal radial fractures in adults? Eur J Orthop Surg Traumatol. 2017 Dec;27(8):1075-1082. doi: 10.1007/s00590-017-2003-z. Epub 2017 Jun 21. PMID: 28638948.

12. Court-Brown CM, Aitken SA, Duckworth AD, Clement ND, McQueen MM. The relationship between social deprivation and the incidence of adult fractures. J Bone Joint Surg Am. 2013 Mar 20;95(6):e321-7. doi: 10.2106/JBJS.K.00631. PMID: 23515993.

13. Fecht D, Jones A, Hill T, Lindfield T, Thomson R, Hansell AL, Shukla R. Inequalities in rural communities: adapting national deprivation indices for rural settings. J Public Health (Oxf). 2018 Jun 1;40(2):419-425. doi: 10.1093/pubmed/fdx048. PMID: 28453666; PMCID: PMC6051444.

14. Jagger DC, Sherriff A, Macpherson LM. Measuring socio-economic inequalities in edentate Scottish adults--cross-sectional analyses using Scottish Health Surveys 1995-2008/09. Community Dent Oral Epidemiol. 2013 Dec;41(6):499-508. doi: 10.1111/cdoe.12040. Epub 2013 Feb 11. PMID: 23398352.

15. Labbe E, Blanquet M, Gerbaud L, Poirier G, Sass C, Vendittelli F, Moulin JJ. A new reliable index to measure individual deprivation: the EPICES score. Eur J Public Health. 2015 Aug;25(4):604-9. doi: 10.1093/eurpub/cku231. Epub 2015 Jan 25. PMID: 25624273.

16. McCormick J, Chen R. Impact of socioeconomic deprivation on mortality in people with haemorrhagic stroke: a population-based cohort study. Postgrad Med J. 2016 Sep;92(1091):501-5. doi: 10.1136/postgradmedj-2015-133663. Epub 2016 Mar 3. PMID: 26941269.

17. Tunstall H, Mitchell R, Pearce J, Shortt N. The general and mental health of movers to more- and less-disadvantaged socio-economic and physical environments within the UK. Soc Sci Med. 2014 Oct;118:97-107. doi: 10.1016/j.socscimed.2014.07.038. Epub 2014 Jul 29. PMID: 25112564.

18. Guio AC, Gordon D, Marlier E, Najera H, Pomati M. Towards an EU measure of child deprivation. Child Indic Res. 2018;11(3):835-860. doi: 10.1007/s12187-017-9491-6. Epub 2017 Oct 10. PMID: 29755609; PMCID: PMC5937875.

19. Bárcena-Martín, Elena & Cuesta, Maite & Rodriguez, Santiago & Moro-Egido, Ana. (2017). Child deprivation and social benefits: Europe in cross-national perspective. Socio-Economic Review. 15. 717-744. 10.1093/ser/mwx019.

20. Meijer M, Engholm G, Grittner U, Bloomfield K. A socioeconomic deprivation index for small areas in Denmark. Scand J Public Health. 2013 Aug;41(6):560-9. doi: 10.1177/1403494813483937. Epub 2013 Apr 18. Erratum in: Scand J Public Health. 2013 Nov;41(7):769. Gritter, Ulrike [corrected to Grittner, Ulrike]. PMID: 23599378.

21. Béjot Y, Bourredjem A, Mimeau E, Joux J, Lannuzel A, Misslin-Tritsch C, Bonithon-Kopp C, Rochemont D, Nacher M, Cabie A, Lalanne Mistrih ML, Fournel I; INDIA Study Group. Social deprivation and 1-year survival after stroke: a prospective cohort study. Eur J Neurol. 2021 Mar;28(3):800-808. doi: 10.1111/ene.14614. Epub 2020 Nov 25. PMID: 33098727.

22. Béjot Y, Guilloteau A, Joux J, Lannuzel A, Mimeau E, Mislin-Tritsch C, Fournel I, Bonithon-Kopp C; INDIA Study Group. Social deprivation and stroke severity on admission: a French cohort study in Burgundy and the West Indies - Guyana region. Eur J Neurol. 2017 May;24(5):694-702. doi: 10.1111/ene.13271. Epub 2017 Feb 25. PMID: 28236340.

23. Bihan H, Cosson E, Khiter C, Vittaz L, Faghfouri F, Leboeuf D, Carbillon L, Dauphin H, Reach G, Valensi P. Factors associated with screening for glucose abnormalities after gestational diabetes mellitus: baseline cohort of the interventional IMPACT study. Diabetes Metab. 2014 Apr;40(2):151-7. doi: 10.1016/j.diabet.2013.12.002. Epub 2014 Feb 3. PMID: 24503190.

24. Blanquet M, Debost-Legrand A, Gerbaud L, de La Celle C, Brigand A, Mioche L, Sass C, Hazart J, Aw A. Metabolic syndrome and social deprivation: results of a French observational multicentre survey. Fam Pract. 2016 Feb;33(1):17-22. doi: 10.1093/fampra/cmv086. Epub 2015 Nov 6. PMID: 26546988.

25. Bongue B, Colvez A, Amsallem E, Gerbaud L, Sass C. Assessment of Health Inequalities Among Older People Using the EPICES Score: A Composite Index of Social Deprivation. J Frailty Aging. 2016;5(3):168-173. PMID: 29240316.

26. Delmas E, Bourredjem A, Nacher M, Cabie A, Mimeau E, Bonithon-Kopp C, Rochemont D, Lalanne-Mistrih ML, Misslin-Tritsch C, Joux J, Lannuzel A, Fournel I, Béjot Y; INDIA Study Group. Impact of Smoking on Functional Prognosis after Ischemic Stroke according to Deprivation: A Prospective Cohort Study. Neuroepidemiology. 2022;56(6):443-451. doi: 10.1159/000526894. Epub 2022 Nov 10. PMID: 36302341.

27. Fouchard A, Bréchat PH, Castiel D, Pascal J, Sass C, Lebas J, Chauvin P. Caractéristiques métrologiques et comparaison de trois outils de repérage de la précarité sociale dans une permanence d’accès aux soins de santé hospitalière à Paris [Qualitative and quantitative comparisons of three individual deprivation scores for outpatients attending a free hospital care clinic in Paris]. Rev Epidemiol Sante Publique. 2014 Aug;62(4):237-47. French. doi: 10.1016/j.respe.2014.04.004. Epub 2014 Jul 11. PMID: 25026886.

28. Furau, Roxana & Popa, Amorin & Socea, Bogdan & Dimitriu, Mihai & Precup, Cris & Furau, Gheorghe & Onel, Mircea & Romosan, Radu-Stefan & Gheroghiu, Diana & Gheroghiu, Nicolae & Furau, Cristian. (2019). Chemical Liaison Between Diabetes and Hypertension, Depression and Socio-economic Deprivation Impact in Both. Revista de Chimie. 70. 2977-2981. 10.37358/RC.19.8.7468.

29. Giovannelli J, Pinon A, Lenain M, Cleys AL, Lefebvre B, Capon N, Spychala S, Boulanger E, Cassagnaud P, Barthoulot M. The relationship between social deprivation and a frailty index of cumulative deficits in French middle-aged caregivers. BMC Geriatr. 2022 Jan 3;22(1):15. doi: 10.1186/s12877-021-02736-3. PMID: 34979976; PMCID: PMC8721985.

30. Goupil de Bouillé J, Collignon M, Capsec J, Guillon L, Le Moal G, Barin F, Roncato M, Hocqueloux L, Stefic K, Bernard L, Gras G. Low-level HIV viremia is associated with low antiretroviral prescription refill rates and social deprivation. AIDS Care. 2021 Nov;33(11):1445-1450. doi: 10.1080/09540121.2020.1806198. Epub 2020 Aug 14. PMID: 32794406.

31. Guilloteau A, Binquet C, Bourredjem A, Fournel I, Lalanne-Mistrih ML, Nacher M, Rochemont D, Cabie A, Mimeau E, Mislin-Tritsch C, Joux J, Lannuzel A, Bonithon-Kopp C, Béjot Y, Devilliers H; INDIA study group. Social deprivation among socio-economic contrasted french areas: Using item response theory analysis to assess differential item functioning of the EPICES questionnaire in stroke patients. PLoS One. 2020 Apr 2;15(4):e0230661. doi: 10.1371/journal.pone.0230661. PMID: 32240217; PMCID: PMC7117693.

32. Gusto G, Vol S, Lasfargues G, Guillaud C, Lantieri O, Tichet J. Deprivation and health risk indicators in full-time permanent workers. Eur J Public Health. 2014 Aug;24(4):585-94. doi: 10.1093/eurpub/ckt138. Epub 2013 Sep 24. PMID: 24068546.

33. Henrotin JB, Vaissière M, Etaix M, Dziurla M, Radauceanu A, Malard S, Lafon D. Deprivation, occupational hazards and perinatal outcomes in pregnant workers. Occup Med (Lond). 2017 Jan;67(1):44-51. doi: 10.1093/occmed/kqw148. Epub 2016 Nov 7. PMID: 27821643.

34. Labbe E, Blanquet M, Gerbaud L, Poirier G, Sass C, Vendittelli F, Moulin JJ. A new reliable index to measure individual deprivation: the EPICES score. Eur J Public Health. 2015 Aug;25(4):604-9. doi: 10.1093/eurpub/cku231. Epub 2015 Jan 25. PMID: 25624273.

35. Lelong A, Jiroff L, Blanquet M, Mourgues C, Leymarie MC, Gerbaud L, Lémery D, Vendittelli F. Is individual social deprivation associated with adverse perinatal outcomes? Results of a French multicentre cross-sectional survey. J Prev Med Hyg. 2015 Aug 5;56(2):E95-E101. PMID: 26789995; PMCID: PMC4718350.

36. Moret L, Anthoine E, Pourreau A, Beaudeau F, Leclère B. Inpatient satisfaction with medical information received from caregivers: an observational study on the effect of social deprivation. BMC Health Serv Res. 2017 Nov 23;17(1):769. doi: 10.1186/s12913-017-2728-8. PMID: 29169348; PMCID: PMC5701506.

37. Raho-Moussa M, Guiguet M, Michaud C, Honoré P, Palacios C, Boué F, Azghay M, Kansau I, Chambrin V, Kandel T, Favier M, Miekoutima E, Sayre N, Pignon C, Shoai M, Bouchaud O, Abgrall S. Respective roles of migration and social deprivation for virological non-suppression in HIV-infected adults on antiretroviral therapy in France. PLoS One. 2019 Mar 7;14(3):e0213019. doi: 10.1371/journal.pone.0213019. PMID: 30845270; PMCID: PMC6405133.

38. Roussel A, Faye A, Lefevre-Utile A, De Pontual L, Chevreul K, Michel M. A comparison of individual and ecological indicators of social deprivation and their association with hospital efficiency in the context of infectious diseases in two French general paediatric departments. Int Health. 2022 Jul 1;14(4):405-412. doi: 10.1093/inthealth/ihz102. PMID: 31990348; PMCID: PMC9248052.

39. Roussel A, Michel M, Lefevre-Utile A, De Pontual L, Faye A, Chevreul K. Impact of social deprivation on length of stay for common infectious diseases in two French university-affiliated general pediatric departments. Arch Pediatr. 2018 Aug;25(6):359-364. doi: 10.1016/j.arcped.2018.06.003. Epub 2018 Jul 21. PMID: 30041884.

40. Zadeh SM, Léger S, Guiguet-Auclair C, Gallot D, Celse MP, Vendittelli F, Debost-Legrand A. Validation of the “EPICES” social deprivation score in a population of women who have just given birth: a French cross-sectional study. Public Health. 2021 Dec;201:19-25. doi: 10.1016/j.puhe.2021.09.027. Epub 2021 Oct 30. PMID: 34742113.

41. Alves A, Civet A, Laurent A, Parc Y, Penna C, Msika S, Hirsch M, Pocard M; Groupe COINCIDE. Social deprivation aggravates post-operative morbidity in carcinologic colorectal surgery: Results of the COINCIDE multicenter study. J Visc Surg. 2021 Jun;158(3):211-219. doi: 10.1016/j.jviscsurg.2020.07.007. Epub 2020 Jul 31. PMID: 32747307.

42. Barry Y, Le Strat Y, Azria E, Gorza M, Pilkington H, Vandentorren S, Gallay A, Regnault N. Ability of municipality-level deprivation indices to capture social inequalities in perinatal health in France: A nationwide study using preterm birth and small for gestational age to illustrate their relevance. BMC Public Health. 2022 May 9;22(1):919. doi: 10.1186/s12889-022-13246-1. PMID: 35534845; PMCID: PMC9082984.

43. Beaumier M, Béchade C, Dejardin O, Lassalle M, Vigneau C, Longlune N, Launay L, Couchoud C, Ficheux M, Lobbedez T, Châtelet V. Is self-care dialysis associated with social deprivation in a universal health care system? A cohort study with data from the Renal Epidemiology and Information Network Registry. Nephrol Dial Transplant. 2020 May 1;35(5):861-869. doi: 10.1093/ndt/gfz245. PMID: 31821495.

44. Beaumier M, Calvar E, Launay L, Béchade C, Lanot A, Schauder N, Touré F, Lassalle M, Couchoud C, Châtelet V, Lobbedez T; REIN registry. Effect of social deprivation on peritoneal dialysis uptake: A mediation analysis with the data of the REIN registry. Perit Dial Int. 2022 Jul;42(4):361-369. doi: 10.1177/08968608211023268. Epub 2021 Jul 1. PMID: 34196237.

45. Belot A, Remontet L, Rachet B, Dejardin O, Charvat H, Bara S, Guizard AV, Roche L, Launoy G, Bossard N. Describing the association between socioeconomic inequalities and cancer survival: methodological guidelines and illustration with population-based data. Clin Epidemiol. 2018 May 17;10:561-573. doi: 10.2147/CLEP.S150848. PMID: 29844706; PMCID: PMC5961638.

46. Berger E, Delpierre C, Despas F, Bertoli S, Bérard E, Bombarde O, Bories P, Sarry A, Laurent G, Récher C, Lamy S. Are social inequalities in acute myeloid leukemia survival explained by differences in treatment utilization? Results from a French longitudinal observational study among older patients. BMC Cancer. 2019 Sep 5;19(1):883. doi: 10.1186/s12885-019-6093-3. PMID: 31488077; PMCID: PMC6729078.

47. Bryere J, Pornet C, Copin N, Launay L, Gusto G, Grosclaude P, Delpierre C, Lang T, Lantieri O, Dejardin O, Launoy G. Assessment of the ecological bias of seven aggregate social deprivation indices. BMC Public Health. 2017 Jan 17;17(1):86. doi: 10.1186/s12889-016-4007-8. PMID: 28095815; PMCID: PMC5240241.

48. Calvar E, Launay L, Bayat-Makoei S, Bauwens M, Lassalle M, Couchoud C, Lobbedez T, Châtelet V. Social deprivation reduced registration for kidney transplantation through markers of nephrological care: a mediation analysis. J Clin Epidemiol. 2023 May;157:92-101. doi: 10.1016/j.jclinepi.2023.03.005. Epub 2023 Mar 10. PMID: 36905970.

49. Calvar E, Launay L, Boyer A, Launoy G, Lobbedez T, Châtelet V. Effects of Social Deprivation on the Proportion of Preemptive Kidney Transplantation: A Mediation Analysis. Transplant Direct. 2021 Sep 7;7(10):e750. doi: 10.1097/TXD.0000000000001203. PMID: 36567853; PMCID: PMC9771216.

50. Châtelet V, Bayat-Makoei S, Vigneau C, Launoy G, Lobbedez T. Renal transplantation outcome and social deprivation in the French healthcare system: a cohort study using the European Deprivation Index. Transpl Int. 2018 Oct;31(10):1089-1098. doi: 10.1111/tri.13161. Epub 2018 Apr 16. PMID: 29611277.

51. Cuzin L, Yazdanpanah Y, Huleux T, Cotte L, Pugliese P, Allavena C, Reynes J, Poizot-Martin I, Bani-Sadr F, Delpierre C; Dat’AIDS Study Group. No relationship between late HIV diagnosis and social deprivation in newly diagnosed patients in France. HIV Med. 2018 Mar;19(3):238-242. doi: 10.1111/hiv.12545. Epub 2017 Aug 18. PMID: 28834136.

52. Driollet B, Bayer F, Chatelet V, Macher MA, Salomon R, Ranchin B, Roussey G, Lahoche A, Garaix F, Decramer S, Mérieau E, Fila M, Zaloszyc A, Deschênes G, Valeri L, Launay L, Couchoud C, Leffondré K, Harambat J. Social deprivation is associated with poor kidney transplantation outcome in children. Kidney Int. 2019 Sep;96(3):769-776. doi: 10.1016/j.kint.2019.05.011. Epub 2019 May 28. PMID: 31375259.

53. Driollet B, Bayer F, Kwon T, Krid S, Ranchin B, Tsimaratos M, Parmentier C, Novo R, Roussey G, Tellier S, Fila M, Zaloszyc A, Godron-Dubrasquet A, Cloarec S, Vrillon I, Broux F, Bérard E, Taque S, Pietrement C, Nobili F, Guigonis V, Launay L, Couchoud C, Harambat J, Leffondré K. Social Deprivation Is Associated With Lower Access to Pre-emptive Kidney Transplantation and More Urgent-Start Dialysis in the Pediatric Population. Kidney Int Rep. 2021 Dec 14;7(4):741-751. doi: 10.1016/j.ekir.2021.12.015. PMID: 35497781; PMCID: PMC9039898.

54. Guillaume E, Pornet C, Dejardin O, Launay L, Lillini R, Vercelli M, Marí-Dell’Olmo M, Fernández Fontelo A, Borrell C, Ribeiro AI, Pina MF, Mayer A, Delpierre C, Rachet B, Launoy G. Development of a cross-cultural deprivation index in five European countries. J Epidemiol Community Health. 2016 May;70(5):493-9. doi: 10.1136/jech-2015-205729. Epub 2015 Dec 11. PMID: 26659762; PMCID: PMC4853548.

55. Lokar K, Zagar T, Zadnik V. Estimation of the Ecological Fallacy in the Geographical Analysis of the Association of Socio-Economic Deprivation and Cancer Incidence. Int J Environ Res Public Health. 2019 Jan 22;16(3):296. doi: 10.3390/ijerph16030296. PMID: 30678244; PMCID: PMC6388200.

56. Merville O, Launay L, Dejardin O, Rollet Q, Bryère J, Guillaume É, Launoy G. Can an Ecological Index of Deprivation Be Used at the Country Level? The Case of the French Version of the European Deprivation Index (F-EDI). Int J Environ Res Public Health. 2022 Feb 17;19(4):2311. doi: 10.3390/ijerph19042311. PMID: 35206501; PMCID: PMC8872283.

57. Michel M, Bryère J, Maravic M, Marcelli C. Knee replacement incidence and social deprivation: results from a French ecological study. Joint Bone Spine. 2019 Oct;86(5):637-641. doi: 10.1016/j.jbspin.2019.03.004. Epub 2019 Mar 22. PMID: 30910705.

58. Morelli X, Rieux C, Cyrys J, Forsberg B, Slama R. Air pollution, health and social deprivation: A fine-scale risk assessment. Environ Res. 2016 May;147:59-70. doi: 10.1016/j.envres.2016.01.030. Epub 2016 Feb 5. Erratum in: Environ Res. 2016 Oct;150:664. doi: 10.1016/j.envres.2016.06.005. PMID: 26852006.

59. Moriceau G, Bourmaud A, Tinquaut F, Oriol M, Jacquin JP, Fournel P, Magné N, Chauvin F. Social inequalities and cancer: can the European deprivation index predict patients’ difficulties in health care access? a pilot study. Oncotarget. 2016 Jan 5;7(1):1055-65. doi: 10.18632/oncotarget.6274. PMID: 26540571; PMCID: PMC4808051.

60. Mulliri A, Gardy J, Dejardin O, Bouvier V, Pocard M, Alves A. Social inequalities in health: How do they influence the natural history of colorectal cancer? J Visc Surg. 2023 Jun;160(3):203-213. doi: 10.1016/j.jviscsurg.2023.02.002. Epub 2023 Apr 14. PMID: 37062638.

61. Ouidir M, Lepeule J, Siroux V, Malherbe L, Meleux F, Rivière E, Launay L, Zaros C, Cheminat M, Charles MA, Slama R. Is atmospheric pollution exposure during pregnancy associated with individual and contextual characteristics? A nationwide study in France. J Epidemiol Community Health. 2017 Oct;71(10):1026-1036. doi: 10.1136/jech-2016-208674. Epub 2017 Aug 22. PMID: 28830952.

62. Perrin A, Freyssenge J, Haesebaert J, Tazarourte K, Termoz A, Grimaud O, Derex L, Viprey M, Schott AM. Are there socio-economic inequities in access to reperfusion therapy: The stroke 69 cohort. Rev Neurol (Paris). 2021 Nov;177(9):1168-1175. doi: 10.1016/j.neurol.2021.02.394. Epub 2021 Jul 15. PMID: 34274130.

63. Petit M-, Bryère J, Maravic M, Pallaro F, Marcelli C. Hip fracture incidence and social deprivation: results from a French ecological study. Osteoporos Int. 2017 Jul;28(7):2045-2051. doi: 10.1007/s00198-017-3998-z. Epub 2017 Mar 23. PMID: 28337523.

64. Ribeiro AI, Launay L, Guillaume E, Launoy G, Barros H. The Portuguese version of the European Deprivation Index: Development and association with all-cause mortality. PLoS One. 2018 Dec 5;13(12):e0208320. doi: 10.1371/journal.pone.0208320. PMID: 30517185; PMCID: PMC6281298.

65. Ribeiro AI, Mayer A, Miranda A, Pina MF. The Portuguese Version of the European Deprivation Index: An Instrument to Study Health Inequalities. Acta Med Port. 2017 Jan 31;30(1):17-25. doi: 10.20344/amp.7387. Epub 2017 Jan 31. PMID: 28501033.

66. Rollet Q, Bouvier V, Launay L, De Mil R, Launoy G, Dejardin O, Alves A. No effect of comorbidities on the association between social deprivation and geographical access to the reference care center in the management of colon cancer. Dig Liver Dis. 2018 Mar;50(3):297-304. doi: 10.1016/j.dld.2017.10.015. Epub 2017 Oct 27. PMID: 29103992.

67. Rollet Q, Guillaume É, Launay L, Launoy G. Socio-Territorial Inequities in the French National Breast Cancer Screening Programme-A Cross-Sectional Multilevel Study. Cancers (Basel). 2021 Aug 30;13(17):4374. doi: 10.3390/cancers13174374. PMID: 34503184; PMCID: PMC8430540.

68. Serman F, Favre J, Deken V, Guittet L, Collins C, Rochoy M, Messaadi N, Duhamel A, Launay L, Berkhout C, Raginel T. The association between cervical cancer screening participation and the deprivation index of the location of the family doctor’s office. PLoS One. 2020 May 15;15(5):e0232814. doi: 10.1371/journal.pone.0232814. PMID: 32413044; PMCID: PMC7228108.

69. Temam S, Varraso R, Pornet C, Sanchez M, Affret A, Jacquemin B, Clavel-Chapelon F, Rey G, Rican S, Le Moual N. Ability of ecological deprivation indices to measure social inequalities in a French cohort. BMC Public Health. 2017 Dec 15;17(1):956. doi: 10.1186/s12889-017-4967-3. PMID: 29246202; PMCID: PMC5732455.

70. Tron L, Remontet L, Fauvernier M, Rachet B, Belot A, Launay L, Merville O, Molinié F, Dejardin O, Francim Group, Launoy G. Is the Social Gradient in Net Survival Observed in France the Result of Inequalities in Cancer-Specific Mortality or Inequalities in General Mortality? Cancers (Basel). 2023 Jan 20;15(3):659. doi: 10.3390/cancers15030659. PMID: 36765616; PMCID: PMC9913401.

71. Vandentorren S, Smaïli S, Chatignoux E, Maurel M, Alleaume C, Neufcourt L, Kelly-Irving M, Delpierre C. The effect of social deprivation on the dynamic of SARS-CoV-2 infection in France: a population-based analysis. Lancet Public Health. 2022 Mar;7(3):e240-e249. doi: 10.1016/S2468-2667(22)00007-X. Epub 2022 Feb 15. PMID: 35176246; PMCID: PMC8843336.

72. Vercelli, M., Lillini, R., Stracci, F. et al. Cancer Mortality and Deprivation: Comparison Among the Performances of the European Deprivation Index, the Italian Deprivation Index and Local Socio-Health Deprivation Indices. Soc Indic Res 151, 599–620 (2020). https://doi.org/10.1007/s11205-020-02396-7.

73. Zadnik V, Guillaume E, Lokar K, Žagar T, Primic Žakelj M, Launoy G, Launay L. Slovenian Version of The European Deprivation Index at Municipal Level. Zdr Varst. 2018 Apr 6;57(2):47-54. doi: 10.2478/sjph-2018-0007. PMID: 29651315; PMCID: PMC5894458.

74. Bertin M, Chevrier C, Pelé F, Serrano-Chavez T, Cordier S, Viel JF. Can a deprivation index be used legitimately over both urban and rural areas? Int J Health Geogr. 2014 Jun 14;13:22. doi: 10.1186/1476-072X-13-22. PMID: 24929662; PMCID: PMC4063986.

75. Bryere J, Pornet C, Copin N, Launay L, Gusto G, Grosclaude P, Delpierre C, Lang T, Lantieri O, Dejardin O, Launoy G. Assessment of the ecological bias of seven aggregate social deprivation indices. BMC Public Health. 2017 Jan 17;17(1):86. doi: 10.1186/s12889-016-4007-8. PMID: 28095815; PMCID: PMC5240241.

76. Barry Y, Le Strat Y, Azria E, Gorza M, Pilkington H, Vandentorren S, Gallay A, Regnault N. Ability of municipality-level deprivation indices to capture social inequalities in perinatal health in France: A nationwide study using preterm birth and small for gestational age to illustrate their relevance. BMC Public Health. 2022 May 9;22(1):919. doi: 10.1186/s12889-022-13246-1. PMID: 35534845; PMCID: PMC9082984.

77. Bastian K, Hollinger A, Mebazaa A, Azoulay E, Féliot E, Chevreul K, Fournier MC, Guidet B, Michel M, Montravers P, Pili-Floury S, Sonneville R, Siegemund M, Gayat E; FROG-ICU Study Investigators. Association of social deprivation with 1-year outcome of ICU survivors: results from the FROG-ICU study. Intensive Care Med. 2018 Dec;44(12):2025-2037. doi: 10.1007/s00134-018-5412-5. Epub 2018 Oct 23. PMID: 30353380; PMCID: PMC7095041.

78. Caudeville, Julien & Rican, Stephane. (2016). Socio-environmental inequality in France: Spatial associations between social deprivation and proximity to potentially hazardous sites. Environnement, Risques et Sante. 15. 39-47. 10.1684/ers.2015.0828.

79. Deborde T, Chatignoux E, Quintin C, Beltzer N, Hamers FF, Rogel A. Breast cancer screening programme participation and socioeconomic deprivation in France. Prev Med. 2018 Oct;115:53-60. doi: 10.1016/j.ypmed.2018.08.006. Epub 2018 Aug 9. PMID: 30099047.

80. Pergeline J, Rivière S, Rey S, Fresson J, Rachas A, Tuppin P. Social deprivation and the use of healthcare services over one year by children less than 18 years of age in 2018: A French nationwide observational study. PLoS One. 2023 May 24;18(5):e0285467. doi: 10.1371/journal.pone.0285467. PMID: 37224152; PMCID: PMC10208476.

81. Yang JS, Michel M, Cogo H, Malorey D, Poey N, Caseris M, Chevreul K, Faye A. Impact of Deprivation on the Incidence and Severity of Tuberculosis in Children: A Retrospective Study from 2007 to 2020 in a Tertiary Care Center in Paris, France. J Pediatr. 2023 Aug;259:113395. doi: 10.1016/j.jpeds.2023.113395. Epub 2023 Mar 29. PMID: 37001636.

82. Temam S, Varraso R, Pornet C, Sanchez M, Affret A, Jacquemin B, Clavel-Chapelon F, Rey G, Rican S, Le Moual N. Ability of ecological deprivation indices to measure social inequalities in a French cohort. BMC Public Health. 2017 Dec 15;17(1):956. doi: 10.1186/s12889-017-4967-3. PMID: 29246202; PMCID: PMC5732455.

83. Tuppin P, Ricci-Renaud P, de Peretti C, Fagot-Campagna A, Alla F, Danchin N, Allemand H. Frequency of cardiovascular diseases and risk factors treated in France according to social deprivation and residence in an overseas territory. Int J Cardiol. 2014 May 15;173(3):430-5. doi: 10.1016/j.ijcard.2014.03.012. Epub 2014 Mar 15. PMID: 24679692.

84. Feuillet T, Valette JF, Charreire H, Kesse-Guyot E, Julia C, Vernez-Moudon A, Hercberg S, Touvier M, Oppert JM. Influence of the urban context on the relationship between neighbourhood deprivation and obesity. Soc Sci Med. 2020 Nov;265:113537. doi: 10.1016/j.socscimed.2020.113537. Epub 2020 Nov 21. PMID: 33250318.

85. Chouaid C, Assié JB, Andujar P, Blein C, Tournier C, Vainchtock A, Scherpereel A, Monnet I, Pairon JC. Determinants of malignant pleural mesothelioma survival and burden of disease in France: a national cohort analysis. Cancer Med. 2018 Apr;7(4):1102-1109. doi: 10.1002/cam4.1378. Epub 2018 Feb 26. PMID: 29479845; PMCID: PMC5911629.

86. Taha MK, Weil-Olivier C, Bouée S, Emery C, Nachbaur G, Pribil C, Loncle-Provot V. Risk factors for invasive meningococcal disease: a retrospective analysis of the French national public health insurance database. Hum Vaccin Immunother. 2021 Jun 3;17(6):1858-1866. doi: 10.1080/21645515.2020.1849518. Epub 2021 Jan 15. PMID: 33449835; PMCID: PMC8115611.

87. Schederecker F, Kurz C, Fairburn J, Maier W. Do alternative weighting approaches for an Index of Multiple Deprivation change the association with mortality? A sensitivity analysis from Germany. BMJ Open. 2019 Aug 26;9(8):e028553. doi: 10.1136/bmjopen-2018-028553. PMID: 31455703; PMCID: PMC6719755.

88. Maier W, Holle R, Hunger M, Peters A, Meisinger C, Greiser KH, Kluttig A, Völzke H, Schipf S, Moebus S, Bokhof B, Berger K, Mueller G, Rathmann W, Tamayo T, Mielck A; DIAB-CORE Consortium. The impact of regional deprivation and individual socio-economic status on the prevalence of Type 2 diabetes in Germany. A pooled analysis of five population-based studies. Diabet Med. 2013 Mar;30(3):e78-86. doi: 10.1111/dme.12062. PMID: 23127142.

89. Hofmeister C, Maier W, Mielck A, Stahl L, Breckenkamp J, Razum O. Regionale Deprivation in Deutschland: Bundesweite Analyse des Zusammenhangs mit Mortalität unter Verwendung des “German Index of Multiple Deprivation (GIMD)” [Regional Deprivation in Germany: Nation-wide Analysis of its Association with Mortality Using the German Index of Multiple Deprivation (GIMD)]. Gesundheitswesen. 2016 Jan;78(1):42-8. German. doi: 10.1055/s-0034-1390421. Epub 2015 Feb 23. PMID: 25706042.

90. Maier, Werner and Schwettmann, Lars. "Regionale Deprivation in Deutschland: Der ‚German Index of Multiple Deprivation (GIMD)‘ " Public Health Forum, vol. 26, no. 4, 2018, pp. 376-379. https://doi.org/10.1515/pubhef-2018-0085.

91. Mena E, Kroll LE, Maier W, Bolte G. Gender inequalities in the association between area deprivation and perceived social support: a cross-sectional multilevel analysis at the municipality level in Germany. BMJ Open. 2018 Apr 12;8(4):e019973. doi: 10.1136/bmjopen-2017-019973. PMID: 29654020; PMCID: PMC5898354.

92. Michalski N, Reis M, Tetzlaff F, Herber M, Kroll LE, Hövener C, Nowossadeck E, Hoebel J. German Index of Socioeconomic Deprivation (GISD): Revision, update and applications. J Health Monit. 2022 Dec 9;7(Suppl 5):2-23. doi: 10.25646/10641. PMID: 36628258; PMCID: PMC9768633.

93. Kroll LE, Schumann M, Hoebel J, Lampert T. Regional health differences - developing a socioeconomic deprivation index for Germany. J Health Monit. 2017 Jun 14;2(2):98-114. doi: 10.17886/RKI-GBE-2017-048.2. PMID: 37152089; PMCID: PMC10161274.

94. Bogner A, Weitz J, Piontek D. The influence of socioeconomic aspects and hospital case volume on survival in colorectal cancer in Saxony, Germany. BMC Cancer. 2023 Mar 10;23(1):228. doi: 10.1186/s12885-023-10672-1. PMID: 36899313; PMCID: PMC9999591.

95. Moissl AP, Delgado GE, Krämer BK, März W, Kleber ME, Grammer TB. Area-based socioeconomic status and mortality: the Ludwigshafen Risk and Cardiovascular Health study. Clin Res Cardiol. 2020 Jan;109(1):103-114. doi: 10.1007/s00392-019-01494-y. Epub 2019 May 29. PMID: 31144063.

96. Conway R, Byrne D, O’Riordan D, Cournane S, Coveney S, Silke B. Influence of social deprivation, overcrowding and family structure on emergency medical admission rates. QJM. 2016 Oct;109(10):675-680. doi: 10.1093/qjmed/hcw053. Epub 2016 Apr 25. PMID: 27118873.

97. Cournane S, Conway R, Byrne D, O’Riordan D, Coveney S, Silke B. Social deprivation and the rate of emergency medical admission for older persons. QJM. 2016 Oct;109(10):645-651. doi: 10.1093/qjmed/hcw029. Epub 2016 Mar 9. PMID: 26966100.

98. Cournane S, Byrne D, Conway R, O’Riordan D, Coveney S, Silke B. Social deprivation and hospital admission rates, length of stay and readmissions in emergency medical admissions. Eur J Intern Med. 2015 Dec;26(10):766-71. doi: 10.1016/j.ejim.2015.09.019. Epub 2015 Oct 21. PMID: 26477015.

99. Conway R, Byrne D, O’Riordan D, Cournane S, Coveney S, Silke B. Deprivation index and dependency ratio are key determinants of emergency medical admission rates. Eur J Intern Med. 2015 Nov;26(9):709-13. doi: 10.1016/j.ejim.2015.09.010. Epub 2015 Sep 26. PMID: 26412675.

100. Cournane S, Dalton A, Byrne D, Conway R, O’Riordan D, Coveney S, Silke B. Social deprivation, population dependency ratio and an extended hospital episode - Insights from acute medicine. Eur J Intern Med. 2015 Nov;26(9):714-9. doi: 10.1016/j.ejim.2015.09.001. Epub 2015 Sep 12. PMID: 26371866.

101. Conway R, O’Riordan D, Byrne D, Cournane S, Coveney S, Silke B. Deprivation influences the emergency admission rate of ambulatory care sensitive conditions. Clin Med (Lond). 2016 Apr;16(2):119-23. doi: 10.7861/clinmedicine.16-2-119. PMID: 27037379; PMCID: PMC4952963.

102. Breslin, Gavin & Fitzpatrick, Ben & Brennan, Deidre & Shannon, Stephen & Rafferty, Ruth & Brien, Wesley & O’Brien, Wesley & Chambers, Fiona & Haughey, Tandy Jane & McCullagh, Darryl & Gormley, Richard & Hanna, Donncha. (2017). Physical activity and wellbeing of 8 to 9 year old children from social disadvantage: An all-Ireland approach to health. Mental Health and Physical Activity. 13. 9-14.

103. Cournane S, Conway R, Byrne D, O’Riordan D, Silke B. Persons with disability, social deprivation and an emergency medical admission. Ir J Med Sci. 2018 Aug;187(3):593-600. doi: 10.1007/s11845-018-1736-y. Epub 2018 Jan 16. PMID: 29340944.

104. Madden JM, More S, Teljeur C, Gleeson J, Walsh C, McGrath G. Population Mobility Trends, Deprivation Index and the Spatio-Temporal Spread of Coronavirus Disease 2019 in Ireland. Int J Environ Res Public Health. 2021 Jun 10;18(12):6285. doi: 10.3390/ijerph18126285. PMID: 34200681; PMCID: PMC8296107.

105. Di Biagio, Katiuscia & Baldini, Marco & Formenti, Luca & Luciani, Aurora & Napolitano, Lara & Sciarra, Iliana & Dolcini, Jacopo & Bartolacci, Silvia & Simeoni, Thomas & Prospero, Emilia. (2020). PROXY EXPOSURE INDICATORS FOR INDOOR AIR POLLUTION, HEALTH IMPACT AND DEPRIVATION IN THE MARCHE REGION, ITALY. Environmental Engineering and Management Journal. 19. 1741-1746. 10.30638/eemj.2020.164.

106. Bartolomeo N, Giotta M, Tafuri S, Trerotoli P. Impact of Socioeconomic Deprivation on the Local Spread of COVID-19 Cases Mediated by the Effect of Seasons and Restrictive Public Health Measures: A Retrospective Observational Study in Apulia Region, Italy. Int J Environ Res Public Health. 2022 Sep 10;19(18):11410. doi: 10.3390/ijerph191811410. PMID: 36141682; PMCID: PMC9517341.

107. Di Filippo A, Perna S, Pierantozzi A, Milozzi F, Fortinguerra F, Caranci N, Moro L, Agabiti N, Belleudi V, Cesaroni G, Nardi A, Spadea T, Gnavi R, Trotta F. Socio-economic inequalities in the use of drugs for the treatment of chronic diseases in Italy. Int J Equity Health. 2022 Nov 9;21(1):157. doi: 10.1186/s12939-022-01772-8. PMID: 36352409; PMCID: PMC9644599.

108. Giotta M, Addabbo F, Mincuzzi A, Bartolomeo N. The Impact of the COVID-19 Pandemic and Socioeconomic Deprivation on Admissions to the Emergency Department for Psychiatric Illness: An Observational Study in a Province of Southern Italy. Life (Basel). 2023 Apr 3;13(4):943. doi: 10.3390/life13040943. PMID: 37109472; PMCID: PMC10143488.

109. Di Giovanni P, Cedrone F, Di Martino G, Romano F, Staniscia T. Paediatric ambulatory care sensitive hospitalisation and Italian deprivation index: retrospective multilevel analysis of administrative data from 2008 to 2018 in the Abruzzo Region (Southern Italy). Epidemiol Prev. 2020 Sep-Dec;44(5-6 Suppl 1):163-169. English. doi: 10.19191/EP20.5-6.S1.P163.086. PMID: 33415959.

110. Vercelli, M., Lillini, R., Stracci, F. et al. Cancer Mortality and Deprivation: Comparison Among the Performances of the European Deprivation Index, the Italian Deprivation Index and Local Socio-Health Deprivation Indices. Soc Indic Res 151, 599–620 (2020). https://doi.org/10.1007/s11205-020-02396-7.

111. Di Salvo F, Caranci N, Spadea T, Zengarini N, Minicozzi P, Amash H, Fusco M, Stracci F, Falcini F, Cirilli C, Candela G, Cusimano R, Tumino R, Sant M; Socioeconomic Inequalities and Oncological Outcomes Italian Working Group. Socioeconomic deprivation worsens the outcomes of Italian women with hormone receptor-positive breast cancer and decreases the possibility of receiving standard care. Oncotarget. 2017 Jul 22;8(40):68402-68414. doi: 10.18632/oncotarget.19447. PMID: 28978126; PMCID: PMC5620266.

112. Rosano A, Pacelli B, Zengarini N, Costa G, Cislaghi C, Caranci N. Aggiornamento e revisione dell’indice di deprivazione italiano 2011 a livello di sezione di censimento [Update and review of the 2011 Italian deprivation index calculated at the census section level]. Epidemiol Prev. 2020 Mar-Jun;44(2-3):162-170. Italian. doi: 10.19191/EP20.2-3.P162.039. PMID: 32631016.

113. Rea F, Ferrante M, Scondotto S, Corrao G. Small-area deprivation index does not improve the capability of multisource comorbidity score in mortality prediction. Front Public Health. 2023 May 16;11:1128377. doi: 10.3389/fpubh.2023.1128377. PMID: 37261238; PMCID: PMC10228715.

114. Minichilli F, Santoro M, Bianchi F, Caranci N, De Santis M, Pasetto R. La valutazione dell’uso dell’indice di deprivazione socioeconomica a livello di area negli studi ecologici su ambiente e salute [Evaluation of the use of the socioeconomic deprivation index at area level in ecological studies on environment and health]. Epidemiol Prev. 2017 May-Aug;41(3-4):187-196. Italian. doi: 10.19191/EP17.3-4.P187.052. PMID: 28929715.

115. Deguen S, Ahlers N, Gilles M, Danzon A, Carayol M, Zmirou-Navier D, Kihal-Talantikite W. Using a Clustering Approach to Investigate Socio-Environmental Inequality in Preterm Birth-A Study Conducted at Fine Spatial Scale in Paris (France). Int J Environ Res Public Health. 2018 Aug 31;15(9):1895. doi: 10.3390/ijerph15091895. PMID: 30200368; PMCID: PMC6163167.

116. Padilla CM, Deguen S, Lalloue B, Blanchard O, Beaugard C, Troude F, Navier DZ, Vieira VM. Cluster analysis of social and environment inequalities of infant mortality. A spatial study in small areas revealed by local disease mapping in France. Sci Total Environ. 2013 Jun 1;454-455:433-41. doi: 10.1016/j.scitotenv.2013.03.027. Epub 2013 Apr 9. PMID: 23563257; PMCID: PMC4097309.

117. Sabel CE, Kihal W, Bard D, Weber C. Creation of synthetic homogeneous neighbourhoods using zone design algorithms to explore relationships between asthma and deprivation in Strasbourg, France. Soc Sci Med. 2013 Aug;91:110-21. doi: 10.1016/j.socscimed.2012.11.018. Epub 2012 Nov 28. PMID: 23332654.

118. Padilla CM, Kihal-Talantikite W, Vieira VM, Rossello P, Le Nir G, Zmirou-Navier D, Deguen S. Air quality and social deprivation in four French metropolitan areas--a localized spatio-temporal environmental inequality analysis. Environ Res. 2014 Oct;134:315-24. doi: 10.1016/j.envres.2014.07.017. Epub 2014 Sep 7. PMID: 25199972; PMCID: PMC4294705.

119. Benmarhnia T, Oulhote Y, Petit C, Lapostolle A, Chauvin P, Zmirou-Navier D, Deguen S. Chronic air pollution and social deprivation as modifiers of the association between high temperature and daily mortality. Environ Health. 2014 Jun 18;13(1):53. doi: 10.1186/1476-069X-13-53. PMID: 24941876; PMCID: PMC4073194.

120. Rooney JP, Tobin K, Crampsie A, Vajda A, Heverin M, McLaughlin R, Staines A, Hardiman O. Social deprivation and population density are not associated with small area risk of amyotrophic lateral sclerosis. Environ Res. 2015 Oct;142:141-7. doi: 10.1016/j.envres.2015.06.024. Epub 2015 Jul 2. PMID: 26142719.

121. O’Farrell IB, Corcoran P, Perry IJ. The area level association between suicide, deprivation, social fragmentation and population density in the Republic of Ireland: a national study. Soc Psychiatry Psychiatr Epidemiol. 2016 Jun;51(6):839-47. doi: 10.1007/s00127-016-1205-8. Epub 2016 Apr 8. PMID: 27059662.

122. Matthews E, Muldoon M, O’Keeffe N, McCarthy KF. Social deprivation and paediatric chronic pain referrals in Ireland: a cross-sectional study. Scand J Pain. 2021 Jun 3;21(3):597-605. doi: 10.1515/sjpain-2021-0031. PMID: 34080402.

123. Swan L, Horgan NF, Fan CW, Warters A, O’Sullivan M. Residential Area Socioeconomic Deprivation is Associated with Physical Dependency and Polypharmacy in Community-Dwelling Older Adults: An Analysis of Health Administrative Data in Ireland. J Multidiscip Healthc. 2022 Sep 2;15:1955-1963. doi: 10.2147/JMDH.S380456. PMID: 36081581; PMCID: PMC9447443.

124. Zelenina A. Russian subject-level index of multidimensional deprivation and its association with all-cause and infant mortality. J Prev Med Hyg. 2022 Dec 31;63(4):E533-E540. doi: 10.15167/2421-4248/jpmh2022.63.4.2498. PMID: 36890998; PMCID: PMC9986986.

125. Vercelli M, Lillini R, Arata L, Zangrillo F, Bagnasco A, Sasso L, Magliani A, Gasparini R, Amicizia D, Panatto D. Analysis of influenza vaccination coverage among the elderly in Genoa (Italy) based on a deprivation index, 2009-2013. J Prev Med Hyg. 2019 Feb 28;59(4 Suppl 2):E11-E17. doi: 10.15167/2421-4248/jpmh2018.59.4s2.1171. PMID: 31016262; PMCID: PMC6419309.

126. Vercelli M, Lillini R. Deindustrialisation, demographic decline, aging, economic crisis and social involution in a metropolitan area analysed by applying Socio-Economic and Health Deprivation Indices. J Prev Med Hyg. 2021 Sep 15;62(3):E709-E717. doi: 10.15167/2421-4248/jpmh2021.62.2.1889. PMID: 34909499; PMCID: PMC8639130.

127. Fortunato F, Iannelli G, Cozza A, Del Prete M, Pollidoro FV, Cocciardi S, DI Trani M, Martinelli D, Prato R. Local deprivation status and seasonal influenza vaccination coverage in adults ≥ 65 years residing in the Foggia municipality, Italy, 2009-2016. J Prev Med Hyg. 2019 Feb 28;59(4 Suppl 2):E51-E64. doi: 10.15167/2421-4248/jpmh2018.59.4s2.1167. PMID: 31016268; PMCID: PMC6419308.

128. Bechini A, Pieralli F, Chellini E, Martini A, Dugheri G, Crescioli F, Scatena T, Baggiani L, Lillini R, Fiaschi P, Bonanni P, Boccalini S. Application of socio-economic-health deprivation index, analysis of mortality and influenza vaccination coverage in the elderly population of Tuscany. J Prev Med Hyg. 2019 Feb 28;59(4 Suppl 2):E18-E25. doi: 10.15167/2421-4248/jpmh2018.59.4s2.1116. PMID: 31016263; PMCID: PMC6419307.

129. Restivo V, Cernigliaro A, Palmeri S, Sinatra I, Costantino C, Casuccio A. The Socio-Economic Health Deprivation Index and its association with mortality and attitudes towards influenza vaccination among the elderly in Palermo, Sicily. J Prev Med Hyg. 2019 Feb 28;59(4 Suppl 2):E26-E30. doi: 10.15167/2421-4248/jpmh2018.59.4s2.1074. PMID: 31016264; PMCID: PMC6419310.

130. Vukovic V, Lillini R, Asta F, Chini F, DE Waure C. Analysis of influenza vaccination coverage among the elderly living in Rome, based on a deprivation index, 2009-2013. J Prev Med Hyg. 2019 Feb 28;59(4 Suppl 2):E31-E37. doi: 10.15167/2421-4248/jpmh2018.59.4s2.1142. PMID: 31016265; PMCID: PMC6419302.

131. Stefanati A, Lupi S, Lillini R, Matteo G, Perrone P, Masetti G, Brosio F, Ferretti S, Gabutti G. Identifying Ferrara’s elderly people with low influenza immunization rates: the contribution of a local socio-economic deprivation index. J Prev Med Hyg. 2019 Feb 28;59(4 Suppl 2):E38-E44. doi: 10.15167/2421-4248/jpmh2018.59.4s2.1033. PMID: 31016266; PMCID: PMC6419304.

132. Hofbauer LM, Rodriguez FS. Validation of a social deprivation index and association with cognitive function and decline in older adults. Int Psychogeriatr. 2021 Dec;33(12):1309-1320. doi: 10.1017/S1041610221000995. Epub 2021 Sep 8. PMID: 34494514.

133. Anderson SG, Shoo H, Saluja S, Anderson CD, Khan A, Livingston M, Jude EB, Lunt M, Dunn G, Heald AH. Social deprivation modifies the association between incident foot ulceration and mortality in type 1 and type 2 diabetes: a longitudinal study of a primary-care cohort. Diabetologia. 2018 Apr;61(4):959-967. doi: 10.1007/s00125-017-4522-x. Epub 2017 Dec 21. PMID: 29264632; PMCID: PMC6448990.

134. Bertin M, Chevrier C, Pelé F, Serrano-Chavez T, Cordier S, Viel JF. Can a deprivation index be used legitimately over both urban and rural areas? Int J Health Geogr. 2014 Jun 14;13:22. doi: 10.1186/1476-072X-13-22. PMID: 24929662; PMCID: PMC4063986.

135. Bryere J, Pornet C, Copin N, Launay L, Gusto G, Grosclaude P, Delpierre C, Lang T, Lantieri O, Dejardin O, Launoy G. Assessment of the ecological bias of seven aggregate social deprivation indices. BMC Public Health. 2017 Jan 17;17(1):86. doi: 10.1186/s12889-016-4007-8. PMID: 28095815; PMCID: PMC5240241.

136. Foster HME, Celis-Morales CA, Nicholl BI, Petermann-Rocha F, Pell JP, Gill JMR, O’Donnell CA, Mair FS. The effect of socioeconomic deprivation on the association between an extended measurement of unhealthy lifestyle factors and health outcomes: a prospective analysis of the UK Biobank cohort. Lancet Public Health. 2018 Dec;3(12):e576-e585. doi: 10.1016/S2468-2667(18)30200-7. Epub 2018 Nov 20. PMID: 30467019.

137. Kendall KM, Bracher-Smith M, Fitzpatrick H, Lynham A, Rees E, Escott-Price V, Owen MJ, O’Donovan MC, Walters JTR, Kirov G. Cognitive performance and functional outcomes of carriers of pathogenic copy number variants: analysis of the UK Biobank. Br J Psychiatry. 2019 May;214(5):297-304. doi: 10.1192/bjp.2018.301. Epub 2019 Feb 15. PMID: 30767844; PMCID: PMC6520248.

138. Klee M, Leist AK, Veldsman M, Ranson JM, Llewellyn DJ. Socioeconomic Deprivation, Genetic Risk, and Incident Dementia. Am J Prev Med. 2023 May;64(5):621-630. doi: 10.1016/j.amepre.2023.01.012. Epub 2023 Mar 6. PMID: 37085245; PMCID: PMC10126314.

139. Labbe E, Blanquet M, Gerbaud L, Poirier G, Sass C, Vendittelli F, Moulin JJ. A new reliable index to measure individual deprivation: the EPICES score. Eur J Public Health. 2015 Aug;25(4):604-9. doi: 10.1093/eurpub/cku231. Epub 2015 Jan 25. PMID: 25624273.

140. Meijer M, Engholm G, Grittner U, Bloomfield K. A socioeconomic deprivation index for small areas in Denmark. Scand J Public Health. 2013 Aug;41(6):560-9. doi: 10.1177/1403494813483937. Epub 2013 Apr 18. Erratum in: Scand J Public Health. 2013 Nov;41(7):769. Gritter, Ulrike [corrected to Grittner, Ulrike]. PMID: 23599378.

141. Morse, Stephen & Vogiatzakis, Ioannis. (2014). Resource Use and Deprivation: Geographical Analysis of the Ecological Footprint and Townsend Index for England. Sustainability. 6. 4749-4771. 10.3390/su6084749.

142. Patel AP, Paranjpe MD, Kathiresan NP, Rivas MA, Khera AV. Race, Socioeconomic Deprivation, and Hospitalization for COVID-19 in English participants of a National Biobank. medRxiv [Preprint]. 2020 May 2:2020.04.27.20082107. doi: 10.1101/2020.04.27.20082107. Update in: Int J Equity Health. 2020 Jul 6;19(1):114. doi: 10.1186/s12939-020-01227-y. PMID: 32511642; PMCID: PMC7276998.

143. Pei YF, Zhang L. Is the Townsend Deprivation Index a Reliable Predictor of Psychiatric Disorders? Biol Psychiatry. 2021 May 1;89(9):839-841. doi: 10.1016/j.biopsych.2021.02.006. PMID: 33858589.

144. Shohaimi S, Boekholdt MS, Luben R, Wareham NJ, Khaw KT. Distribution of lipid parameters according to different socio-economic indicators- the EPIC-Norfolk prospective population study. BMC Public Health. 2014 Aug 28;14:782. doi: 10.1186/1471-2458-14-782. PMID: 25179437; PMCID: PMC4155077.

145. Temam S, Varraso R, Pornet C, Sanchez M, Affret A, Jacquemin B, Clavel-Chapelon F, Rey G, Rican S, Le Moual N. Ability of ecological deprivation indices to measure social inequalities in a French cohort. BMC Public Health. 2017 Dec 15;17(1):956. doi: 10.1186/s12889-017-4967-3. PMID: 29246202; PMCID: PMC5732455.

146. Tyrrell J, Wood AR, Ames RM, Yaghootkar H, Beaumont RN, Jones SE, Tuke MA, Ruth KS, Freathy RM, Davey Smith G, Joost S, Guessous I, Murray A, Strachan DP, Kutalik Z, Weedon MN, Frayling TM. Gene-obesogenic environment interactions in the UK Biobank study. Int J Epidemiol. 2017 Apr 1;46(2):559-575. doi: 10.1093/ije/dyw337. PMID: 28073954; PMCID: PMC5837271.

147. Wang C, Zhang X, Li B, Mu D. A study of factors impacting disease based on the Charlson Comorbidity Index in UK Biobank. Front Public Health. 2023 Jan 9;10:1050129. doi: 10.3389/fpubh.2022.1050129. PMID: 36699869; PMCID: PMC9868818.

148. Woodward M, Peters SAE, Harris K. Social deprivation as a risk factor for COVID-19 mortality among women and men in the UK Biobank: nature of risk and context suggests that social interventions are essential to mitigate the effects of future pandemics. J Epidemiol Community Health. 2021 Nov;75(11):1050-1055. doi: 10.1136/jech-2020-215810. Epub 2021 Apr 27. PMID: 33906905; PMCID: PMC8098299.

149. Ye J, Wen Y, Sun X, Chu X, Li P, Cheng B, Cheng S, Liu L, Zhang L, Ma M, Qi X, Liang C, Kafle OP, Jia Y, Wu C, Wang S, Wang X, Ning Y, Sun S, Zhang F. Socioeconomic Deprivation Index Is Associated With Psychiatric Disorders: An Observational and Genome-wide Gene-by-Environment Interaction Analysis in the UK Biobank Cohort. Biol Psychiatry. 2021 May 1;89(9):888-895. doi: 10.1016/j.biopsych.2020.11.019. Epub 2020 Nov 26. PMID: 33500177.

150. Zelenina A, Shalnova S, Maksimov S, Drapkina O. Characteristics of Composite Deprivation Indices Used in Public Health: A Scoping Review Protocol. Int J Environ Res Public Health. 2022 Aug 24;19(17):10565. doi: 10.3390/ijerph191710565. PMID: 36078280; PMCID: PMC9518044.

151. Ciacci, Andrea & Tagliafico, Giulia. (2020). Measuring the Existence of a Link between Crime and Social Deprivation within a Metropolitan Area. Revista de Estudios Andaluces. 40. 192-194. 10.12795/rea.2020.i40.04.

152. Álvarez-del Arco D, Vicente Sánchez M, Alejos B, Pascual C, Regidor E. Construcción de un índice de privación para los barrios de Madrid y Barcelona [Process and results of constructing a deprivation index for the districts of Madrid and Barcelona, Spain]. Rev Esp Salud Publica. 2013 Jul-Aug;87(4):317-29. Spanish. doi: 10.4321/S1135-57272013000400003. PMID: 24100771.

153. Compés Dea ML, Olivan Bellido E, Feja Solana C, Aguilar Palacio I, García-Carpintero Romero Del Hombrebueno G, Adiego Sancho B. Construcción de un índice de privación por zona básica de salud en Aragón a partir de datos de censo de 2011 [Construction of a deprivation index by Basic Healthcare Area in Aragon using Population and Housing Census 2011]. Rev Esp Salud Publica. 2018 Dec 10;92:e201812087. Spanish. PMID: 30531710; PMCID: PMC11587363.

154. Moussaoui S, Chauvin P, Ibanez G, Soler M, Nael V, Morgand C, Robert S. Construction and Validation of an Individual Deprivation Index: a Study Based on a Representative Cohort of the Paris Metropolitan Area. J Urban Health. 2022 Dec;99(6):1170-1182. doi: 10.1007/s11524-022-00648-0. Epub 2022 Jun 2. PMID: 35653078; PMCID: PMC9161768.

155. Lamnisos D, Lambrianidou G, Middleton N. Small-area socioeconomic deprivation indices in Cyprus: development and association with premature mortality. BMC Public Health. 2019 May 22;19(1):627. doi: 10.1186/s12889-019-6973-0. PMID: 31118020; PMCID: PMC6532164.

156. Betti, Gianni & Gagliardi, Francesca & Lemmi, Achille & Verma, Vijay. (2015). Comparative measures of multidimensional deprivation in the European Union. Empirical Economics. 49. 10.1007/s00181-014-0904-9.

157. Bruzzi C, Ivaldi E, Landi S. Non-compensatory aggregation method to measure social and material deprivation in an urban area: relationship with premature mortality. Eur J Health Econ. 2020 Apr;21(3):381-396. doi: 10.1007/s10198-019-01139-x. Epub 2019 Dec 6. PMID: 31811513.

158. Duque I, Domínguez-Berjón MF, Cebrecos A, Prieto-Salceda MD, Esnaola S, Calvo Sánchez M, Marí-Dell’Olmo M; en nombre del Grupo de Determinantes Sociales de la Salud, iniciativa contexto de la Sociedad Española de Epidemiología. Índice de privación en España por sección censal en 2011 [Deprivation index by enumeration district in Spain, 2011]. Gac Sanit. 2021 Mar-Apr;35(2):113-122. Spanish. doi: 10.1016/j.gaceta.2019.10.008. Epub 2020 Feb 1. PMID: 32014314.

159. Jansen L, Eberle A, Emrich K, Gondos A, Holleczek B, Kajüter H, Maier W, Nennecke A, Pritzkuleit R, Brenner H; GEKID Cancer Survival Working Group. Socioeconomic deprivation and cancer survival in Germany: an ecological analysis in 200 districts in Germany. Int J Cancer. 2014 Jun 15;134(12):2951-60. doi: 10.1002/ijc.28624. Epub 2013 Dec 2. PMID: 24259308.

160. Lanza, Giovanna & De Martino, Mattia. (2022). Urban Housing Inequity: Housing Deprivation and Social Response in the City of Naples. Sustainability. 14. 1047. 10.3390/su14031047.

161. Monica, Raileanu & Fusco, Alessio. (2009). Item response theory and the measurement of deprivation: Evidence from PSELL-3. IRISS at CEPS/INSTEAD, IRISS Working Paper Series. 47. 10.1007/s11135-011-9607-x.

162. Quaglia A, Lillini R, Mamo C, Ivaldi E, Vercelli M; SEIH (Socio-Economic Indicators, Health) Working Group. Socio-economic inequalities: a review of methodological issues and the relationships with cancer survival. Crit Rev Oncol Hematol. 2013 Mar;85(3):266-77. doi: 10.1016/j.critrevonc.2012.08.007. Epub 2012 Sep 20. PMID: 22999326.

163. Strömberg U, Baigi A, Holmén A, Parkes BL, Bonander C, Piel FB. A comparison of small-area deprivation indicators for public-health surveillance in Sweden. Scand J Public Health. 2023 Jun;51(4):520-526. doi: 10.1177/14034948211030353. Epub 2021 Jul 20. PMID: 34282665; PMCID: PMC10259086.
