# Supplementary material for: A scoping review of multiple deprivation indices in Europe
Source: Eur J Public Health. 2025 Oct 30;35(6):1122–8. doi: 10.1093/eurpub/ckaf190 (PMC12707476; doi:10.1093/eurpub/ckaf190)
Supplement: ckaf190_Supplementary_Data [file ckaf190_supplementary_data.zip › ejph-2025-01-om-0031-File007.docx]

**Additional file 2 : indicators list per domain**

**(Un)employment / occupation**

1. % manual workers : percentage of people aged 16 years or over employed in sectors services, agriculture, fishing, craftwork, skilled workers in manufacturing industries, construction, mining, installations operators, and non-skilled workers; with respect to the total employed (ABDI)
2. % unemployment : percentage of people aged 16 years or over without a job (unemployed and those seeking work for the first time), with respect to the total economically active population (ABDI)
3. % eventual employees : percentage of people aged 16 or older occupied as eventual employees (ABDI)
4. Unemployment rate (BMID, GIMD, Irish National Deprivation Index, Neighbourhood Deprivation Index)
5. Male unemployment : proportion of economically active males seeking or waiting to start work (Carstairs)
6. PI* on unemployment benefits (DANDEX)
7. PI with manual profession (DANDEX)
8. Unskilled worker-farm worker : occupational classes in 10 categories (1 ‘farmers’ 2 ‘craftsmen-storekeepers’ 3 ‘business leaders-company managers’ 4 ‘intermediate professions-technicians’ 5 ‘employees’ 6 ‘skilled workers’ 7 ‘unskilled workers - farm workers’ 8 ‘former managers’ 9 ‘other retired people’ 10 ‘other non-working individuals (unemployed persons and others)’. (EDI)
9. Unemployment : employment status (employment/unemployment/retired/other non-working individuals) (EDI)
10. Percentage of blue-collar workers in the labour force (FDEP, Neighbourhood Deprivation Index)
11. Percentage of unemployed individuals aged 15–64 in the active population (FDEP)
12. Unemployment rate : proportion of unemployed as share of inhabitants of working age (GISD)
13. Employment rate : employees subject to social insurance contributions at place of residence per 100 inhabitants of working age (GISD)
14. Gross wage and salary : monthly gross earnings of employees in EUR (GISD)
15. Proportion of working-age population claiming benefits due to incapacity to work (GIMD)
16. % of working population unemployed or seeking first job (IDI)
17. % people with insecure job (Neighbourhood Deprivation Index)
18. % white collar (Neighbourhood Deprivation Index)
19. % self-employed people (Neighbourhood Deprivation Index)
20. % managers workers (Neighbourhood Deprivation Index)
21. Percentage of households headed by professionals or managerial and technical employees, including farmers with 100 acres or more (Pobal HP)
22. Percentage of households headed by semi-skilled or unskilled manual workers, including farmers with less than 30 acres (Pobal HP)
23. Male unemployment rate (Pobal HP)
24. Female unemployment rate (Pobal HP)
25. Unemployment rate : population 15 or older unemployed (Russian deprivation index)
26. Job stability (SoDep)
27. Proportion of economically active residents who are unemployed (Townsend Deprivation Index)

**Education**

1. % insufficient instruction : percentage of people aged 16 years and over who, according to a list of the National Statistics Institute, cannot read or write; can read and write but have less than 5 years schooling; went to school for 5 years or more but did not complete basic compulsory education, with respect to the total population aged 16 years and over (ABDI)
2. % insufficient level of education (Composed socioeconomic index)
3. % young insufficient instruction : percentage of people aged between 16 and 29 years with low educational level, with respect to the total population aged from 16 to 29 years (ABDI)
4. % lack of vocational training (BMID)
5. PI with basic education (DANDEX)
6. Low level of education : ‘less than first stage of secondary-level education’, versus ‘all other levels of education’ (EDI, Neighbourhood Deprivation Index)
7. Percentage of high school graduates in the population ≥15 y of age (FDEP, Neighbourhood Deprivation Index)
8. Employees with university degree : proportion of employees subject to social insurance contributions at place of residence with university degree as share of total employees subject to social insurance contributions at place of residence in % (GISD)
9. Employees without qualification : proportion of employees subject to social insurance contributions at place of residence without professional qualification as share of total employees subject to social insurance contributions at place of residence in % (GISD)
10. School leavers without qualification : proportion of school leavers without lower secondary school leavers certificate out of all school leavers in % (GISD, GIMD)
11. Proportion of individuals without a vocational qualification (GIMD)
12. % of population with education at or below elementary school leaving certificate (IDI)
13. Percentage of population with a primary school education only (Pobal HP)
14. Percentage of population with a third level education (Pobal HP, SEHDI)
15. Education years (SoDep)

**Nationality**

1. % recent foreigners born in low income country : percentage of population born in countries other than the following: Spain, Austria, Belgium, Cyprus, Denmark, Finland, France, Germany, Greece, Ireland, Iceland, Italy, Liechtenstein, Luxembourg, Malta, Monaco, Netherlands, Norway, Portugal, Andorra, Germany, San Marino, Vatican City, Sweden, Switzerland, Canada, United States of America, Japan, Australia and New Zealand; respect to the total population (ABDI)
2. % foreigners whose father or mother born in low income country : percentage of population born or with their father or mother born in countries other than listed above, respect to the total population.(ABDI)
3. Foreign nationality: ‘Foreign nationality’ versus ‘French nationality’. And six unordered variables with two or more categories (EDI)
4. % immigrants (Neighbourhood Deprivation Index)
5. % foreigners (Neighbourhood Deprivation Index)

**Income**

1. % financial poverty of residents (BMID)
2. financial situation of the districts (BMID)
3. low social class : proportion of all persons in private households with an economically active head of household in social class IV or V (Carstairs)
4. Low social class (Irish National Deprivation Index)
5. PI with low disposable income (DANDEX)
6. Median income per household (FDEP, Neighbourhood Deprivation Index)
7. Net household income : average household income in € per inhabitant (GISD, GIMD)
8. Debot quota : private debtors per 100 inhabitants aged 18 and above in % (GISD)
9. Tax revenue : income tax in € per inhabitant (GISD)
10. Low income : percentage of people below a low income threshold in the total population (Russian deprivation index)
11. Household’s net financial assets less than household’s financial liabilities (SoDep)

**Social capital**

1. % lack of social capital (BMID)
2. Do you ever meet a social worker? (EPICES)
3. In the last 6 months, have you had contact with family members other than your parents or children? (EPICES)
4. In the event of difficulties (financial, family, health...), is there anyone in your circle you can count on to put you up for a few days in case of need? (EPICES)
5. In the event of diffi culty (fi nancial, family, health...), is there anyone in your entourage you can count on to provide you with material assistance (including a loan)?material assistance (including loans)? (EPICES)

**Environment**

1. % poor quality of the physical environment) (BMID)
2. Nitrogen dioxide (thousand tons) from stationary sources (Russian deprivation index)
3. Sulphur dioxide (thousand tons) from stationary sources (Russian deprivation index)
4. Carbon monoxide (thousand tons) from stationary sources (Russian deprivation index)
5. Air emissions from vehicle (thousand tons) (Russian deprivation index)
6. The number of fire forest incidence (unit) ((Russian deprivation index)
7. The area of dead forest (hectares) (Russian deprivation index)

**Security/crime**

1. % accidents and crime rates (BMID)
2. PI* with criminal record (DANDEX)
3. Crime rate (number of criminal offenses per population) (GIMD)
4. The number of recorded environmental crimes (Russian deprivation index)

**Material**

1. Lack of car ownership : proportion of all persons in private households which do not own a car (Carstairs)
2. PH** with no car (Child Material and Social deprivation, DANDEX, EDI, Irish National Deprivation Index, Neighbourhood Deprivation Index, Townsend Deprivation Index)
3. Household with 2 or more cars (Neighbourhood Deprivation Index)
4. Child: Some new (not second-hand) clothes - (inability for the person to: replace worn-out clothes with some new ones, have two pairs of properly fitting shoes, spend a small amount of money each week on him/herself, have regular leisure activities, get together with friends/family for a drink/ meal at least once a month, and have an internet connection (Child Material and Social deprivation)
5. Child: Two pairs of properly fitting shoes (Child Material and Social deprivation)
6. Child: Fresh fruits and vegetables daily - cannot afford a meal with meat, chicken, fish or vegetarian equivalent every second day (Child Material and Social deprivation)
7. Child: Meat, chicken, fish or vegetarian equivalent daily (Child Material and Social deprivation)
8. Child: Books at home suitable for the children’s age (Child Material and Social deprivation)
9. Child: Outdoor leisure equipment (Child Material and Social deprivation)
10. Child: Indoor games (Child Material and Social deprivation)
11. Child: Suitable place to do homework (Child Material and Social deprivation)
12. Child: Regular leisure activities (Child Material and Social deprivation)
13. Child: Celebrations on special occasions (Child Material and Social deprivation)
14. Child: Invitation of friends to play and eat from time to time (Child Material and Social deprivation)
15. Child: Participation in school trips and school events that cost money (Child Material and Social deprivation)
16. Child: Holiday – cannot afford one week annual holiday away from home (Child Material and Social deprivation)
17. Household : replace worn-out furniture (Child Material and Social deprivation)
18. Have you been to a show (cinema, theater...) in the last 12 months? (EPICES)
19. Have you been on vacation in the last 12 months? (EPICES)
20. Phone : percentage of households with telephone (Russian deprivation index)

**Overcrowding**

1. Proportion of all persons living in private households with a density of more than one person per room (Carstairs)
2. PH** with more than one person per room (DANDEX, EDI, Townsend Deprivation Index)
3. Overcrowded : percentage of households (individual (singlefamily) houses, individual and communal apartments) with > 5 persons (Russian deprivation index)

**Basic amenities**

1. Household : home adequately warm - cannot keep their homes adequately warm (Child Material and Social deprivation)
2. Heating: ‘No access to a system of central or electric heating’ versus ‘access to a system of central or electric heating’ (EDI).
3. Adults in the household: Access to internet (Child Material and Social deprivation)
4. Are there any times of the month when you have real financial difficulties to meet your needs (food, rent, electricity, etc.)? (EPICES)
5. Stove heating : percentage of households with stove heating (Russian deprivation index)
6. No hot water supply : percentage of households without heat water supply (Russian deprivation index)
7. No central sewerage system : percentage of households with toilets emptying into a cesspit (Russian deprivation index)
8. No sewerage system : percentage of households without sewage system (Russian deprivation index)
9. % of house with very small kitchen or kitchenette (SEHDI)
10. % houses with bath or shower (SEHDI)

**Housing**

1. PH** being rented (DANDEX, IDI)
2. Are you a homeowner (or first-time buyer)? (EPICES)
3. Non owner : tenure (owners/renters at market prices/renters in low-rent community housing/accommodated free of charge) (EDI)
4. Housing density (occupants per 100 m²) (IDI)
5. % subsidized house among all primary residences (Neighbourhood Deprivation Index)
6. Non-owner occupying primary residence (Irish National Deprivation Index, Neighbourhood Deprivation Index, Townsend Deprivation Index)
7. Individual house as a primary residence (Neighbourhood Deprivation Index)
8. Apartment building as a primary residence (Neighbourhood Deprivation Index)
9. Primary residence with a minimum surface area of 100m² (Neighbourhood Deprivation Index)
10. Primary residence with a garage or other parking space (Neighbourhood Deprivation Index)
11. Mean number of persons per room (Pobal HP)

**Family structure**

1. PI* living alone with child/children (DANDEX, IDI, Neighbourhood Deprivation Index, SEHDI)
2. Do you live with a partner? (EPICES)
3. Single-parent household : household types (single pensioner/single-parent household/ couple without children/couple with child(ren)/without family) (EDI)
4. Household with 6+ persons : number of persons in household (≥2 persons/ ≥3 persons/ ≥4 persons/ ≥5 persons/ ≥6 persons) (EDI)
5. Proportion of single-person households (GIMD)
6. Percentage of households with children aged under 15 years and headed by a single parent (Pobal HP)
7. Children +3 : Percentage of families with 3 and more children (ages 0-18) (Russian deprivation index)
8. Average N of people per family (SEHDI)

**Demography**

1. People aged 25 years or younger (Neighbourhood Deprivation Index)
2. Percentage change in population over the previous five years (Pobal HP)
3. percentage of population aged under 15 or over 64 years of age (Pobal HP)
4. Children under 5 years old : Children ages 0-4 as a percentage of total population (Russian deprivation index)
5. Old-age index (SEHDI)
6. % widowers/widow (SEHDI)
7. % married or % separated and divorced (SEHDI)
8. % single-member families aged 65+ (SEHDI)

**Others**

1. Household : Arrears - cannot avoid arrears (Child Material and Social deprivation)
2. Do you have supplementary health insurance ? (EPICES)
3. Have you exercised in the last 12 months? (EPICES)
4. Health insurance status (SoDep)

*PI = proportion inhabitants

**PH = proportion of households
